# Genomic analysis of hypoxia inducible factor alpha in ray-finned fishes reveals missing Ohnologs and evidence of widespread positive selection

**DOI:** 10.1038/s41598-022-26876-7

**Published:** 2022-12-24

**Authors:** Ian K. Townley, Courtney H. Babin, Taylor E. Murphy, Christopher M. Summa, Bernard B. Rees

**Affiliations:** 1 Science Department, Saint George’s School, Spokane, WA 99208 USA; 2 Department of Biological Sciences, University of New Orleans, New Orleans, LA 70148 USA; 3 Department of Computer Sciences, University of New Orleans, New Orleans, LA 70148 USA

**Keywords:** Molecular evolution, Comparative genomics

## Abstract

As aquatic hypoxia worsens on a global scale, fishes will become increasingly challenged by low oxygen, and understanding the molecular basis of their response to hypoxia may help to better define the capacity of fishes to cope with this challenge. The hypoxia inducible factor (HIF) plays a critical role in the molecular response to hypoxia by activating the transcription of genes that serve to improve oxygen delivery to the tissues or enhance the capacity of tissues to function at low oxygen. The current study examines the molecular evolution of genes encoding the oxygen-dependent HIFα subunit (*HIFA*) in the ray-finned fishes (Actinopterygii). Genomic analyses demonstrate that several lineages retain four paralogs of *HIFA* predicted from two rounds of genome duplication at the base of vertebrate evolution, broaden the known distribution of teleost-specific *HIFA* paralogs, and provide evidence for salmonid-specific *HIFA* duplicates. Evolution of the *HIFA* gene family is characterized by widespread episodic positive selection at amino acid sites that potentially mediate protein stability, protein–protein interactions, and transcriptional regulation. *HIFA* transcript abundance depends upon paralog, tissue, and fish lineage. A phylogenetically-informed gene nomenclature is proposed along with avenues for future research on this critical family of transcription factors.

## Introduction

Aquatic hypoxia (low oxygen) varies in spatial scope, severity, and frequency, and it is increasing globally due to human activities (e.g., climate change, eutrophication)^1^. Oxygen availability has been proposed to be a major determinant of the distribution of marine animals^2,3^, and, along with changes in temperature and pH, hypoxia is widely recognized as a significant threat to aquatic organisms^1–3^. The importance of oxygen arises from its central role in aerobic energy metabolism, which supports critical cellular and organismal functions, including ion transport, motility, growth, and reproduction. Hence, oxygen is essential for the normal physiological function of most metazoans, and complex regulatory mechanisms have evolved to mitigate the deleterious effects of hypoxia.

The molecular responses to low oxygen are orchestrated by the hypoxia inducible factor (HIF) family of transcription factors^4,5^. The HIF transcription factor is comprised of two non-identical protein subunits (α and β), both of which are members of the basic helix-loop-helix Per-ARNT-Sim (bHLH-PAS) family of transcription factors^6,7^. The HIFβ subunit, also known as the aryl hydrocarbon receptor nuclear translocator (ARNT), is constitutively expressed, oxygen-independent, and serves other roles in cell signaling^7^. The oxygen-dependence of HIF function is attributed to an increase in the cellular concentration of the HIFα subunit during hypoxia, driven largely by a decrease in the rate of its degradation. At normal oxygen levels (normoxia), specific proline residues of the HIFα subunit are modified by hydroxylation, which signals the protein for ubiquitin-dependent degradation^8–10^. During hypoxia, proline hydroxylation and protein degradation of the HIFα subunit are blocked, whereupon HIFα accumulates, dimerizes with HIFβ, translocates to the nucleus, and, together with accessory proteins, binds specific DNA elements in target genes and activates their transcription. Gene targets of HIF number in the hundreds and include genes involved in oxygen transport, glucose uptake and metabolism, and cell survival and proliferation^11^. These molecular responses serve to ensure oxygen delivery to tissues or enhance the function of tissues during hypoxia.

HIF has been studied extensively in humans, where low tissue oxygenation is associated with several pathologies, including cardiovascular disease, pulmonary disease, and cancer, but also with fetal development and exposure to high-altitude hypoxia^4,5,12–15^. In humans, as in other mammals, three genes encode different HIFα subunits, HIF1α (*HIF1A*), HIF2α (*HIF2A*), and HIF3α (*HIF3A*; see Table 1), which likely arose from two rounds of genome duplication at the base of vertebrate evolution^16^. The protein products dimerize with HIFβ to form the active transcription factors, HIF1, HIF2, and HIF3. Among these, HIF1 is the most well-characterized, has the broadest tissue distribution and gene specificity, and is essential for proper development and the response to hypoxia in mammals^5^. HIF2, initially characterized in endothelial tissues and also known as endothelial PAS protein 1 (EPAS1), is more restricted with respect to tissue distribution and target genes, some of which are shared with HIF1^12,17^. It is critical for angiogenesis, cancer progression, and high-altitude adaptation in humans^12,14,15,18^. HIF3 is the least well-described. Like HIF1α and HIF2α, HIF3α dimerizes with HIFβ to regulate the expression of specific genes; however, a variety of shortened forms, translated from splice variants, act as negative regulators of HIF1α or HIF2α^19–21^.

**Table 1:** Nomenclature conventions and synonyms for the hypoxia inducible factor.

Example	Rule
*HIF* *hif*	The Human Genome Consortium Gene Nomenclature Committee specifies uppercase Latin letters be used for gene symbols. The Zebrafish Nomenclature Conventions specify lowercase Latin letters for gene symbols
*1,2,3,4*	Paralogs that arose during two rounds of genome duplication at the base of vertebrate evolution are indicated by Arabic numbers^a^
*A* or *B* *a* or *b*	The gene encoding the α and β subunits are distinguished by uppercase (humans) or lowercase (zebrafish) Latin letters^b^
*a* or *b*	Paralogs that arose from the teleost-specific genome duplication are indicated by lowercase Latin letters

The gene symbols for hypoxia inducible factors generally have four parts, corresponding to the gene name, the vertebrate-specific paralog, whether it encodes an α or β subunit, and the teleost-specific paralog (for fishes). This study follows the conventions for humans because it has been broadly applied to other vertebrates and invertebrates^31^.

^a^The hypoxia inducible factor 2 alpha subunit is also known as the endothelial PAS-domain protein, *EPAS* in humans and *epas* in zebrafish.

^b^The hypoxia inducible factor β subunit is also known as the aryl hydrocarbon receptor nuclear translocator, *ARNT* in humans and *arnt* in zebrafish.

The ray-finned fishes (Actinopterygii) are the most speciose and diverse class of vertebrates, having over 30,000 species occupying virtually every aquatic habitat on earth^22^. Understanding HIF signaling among fishes could provide insights into their evolutionary history and their capacity to respond to the increasing prevalence of aquatic hypoxia. The initial characterization of HIFα subunits in fishes demonstrated that they possessed orthologs of the three genes found in mammals (reviewed in^23–25^). In a comprehensive phylogenetic analysis of *HIFA* genes in fishes, Rytkonen et al.^26^ found evidence that certain fish lineages retained duplicated copies of the *HIFA* genes that presumably arose from another round of genome duplication, the teleost-specific genome duplication (TGD)^27,28^. Specifically, the family Cyprinidae (carp and its allies, including the zebrafish, *Danio rerio*) was proposed to have teleost-specific paralogs *HIF1Aa/b*, *HIF2Aa/b*, and *HIF3Aa/b* (see Table 1). While these teleost-specific paralogs appear to be lost in most other lineages of fish, Rytkonen et al.^26^ present evidence that certain species among the more-derived Neoteleostei retained a shortened duplicate of *HIF2Ab*, a putative “relic” of the TGD.

Over the last decade, sequence analyses based primarily on *HIFA* transcripts from various fishes generally support the conclusions of Rytkonen et al.^26^; however, several questions regarding the evolution of the *HIFA* gene family remain unanswered. For example, the two rounds of genome duplication at the base of vertebrate evolution are predicted to result in four *HIFA* paralogs (“Ohnologs”^29^), rather than the three paralogs generally recognized to exist in vertebrates. While it is possible that nonfunctionalization of one paralog after the second round of genome duplication^30^ could account for this “missing” Ohnolog^31^, recent analyses have demonstrated that several fishes have *HIF-like*, *HIFA-like*, and *HIF1A-like* genes^32^. The relationships of these genes to one another and to the other *HIFA* genes, however, have not been resolved. Furthermore, the relationships among teleost-specific paralogs and their broader distribution among fishes are not well-established. This is especially true of the “relic” *HIF2Ab*. These uncertain relationships within and among teleost-specific paralogs has led to an inconsistent nomenclature of *HIFA* paralogs. Also, certain fish lineages have undergone additional rounds of genome duplication, for example the salmonid-specific genome duplication (SGD)^33,34^, which potentially further increased the diversity of *HIFA* genes in those lineages. Finally, there has been no systematic evaluation of the tissue expression of *HIFA* transcripts among fishes, which could help to clarify the contribution of subfunctionalization and neofunctionalization^35^ to the maintenance of *HIFA* paralogs.

The current study reexamines the evolution of *HIFA* in ray-finned fishes using recently sequenced genomes, including that of spotted gar (*Lepisosteus oculatus*) to represent a lineage that diverged prior to the TGD^36^. Specifically, we sought to determine (1) whether any ray-finned fishes retained the four *HIFA* genes that arose during the two rounds of genome duplication at the base of vertebrate evolution, (2) whether gene duplicates arising from the TGD are seen in fishes other than the Cyprinidae, (3) the distribution and phylogenetic relationship of shortened forms of *HIF2Ab*, (4) whether *HIFA* duplicates from the SGD are present in salmonid genomes, (5) the potential modes of selection acting on *HIFA* genes and the corresponding amino acid sites potentially under selection, and (6) the broad patterns of tissue expression of *HIFA* transcripts. This analysis of *HIFA* evolution in the ray-finned fishes provides evidence of “missing” Ohnologs, clarifies the relationships among teleost-specific paralogs, provides insights into the selective forces responsible for this diversity, and forwards a recommendation for a phylogenetically-based *HIFA* nomenclature.

## Results

### Evidence for a “Missing” *HIFA* Ohnolog in ray-finned fishes

**Figure 1:**
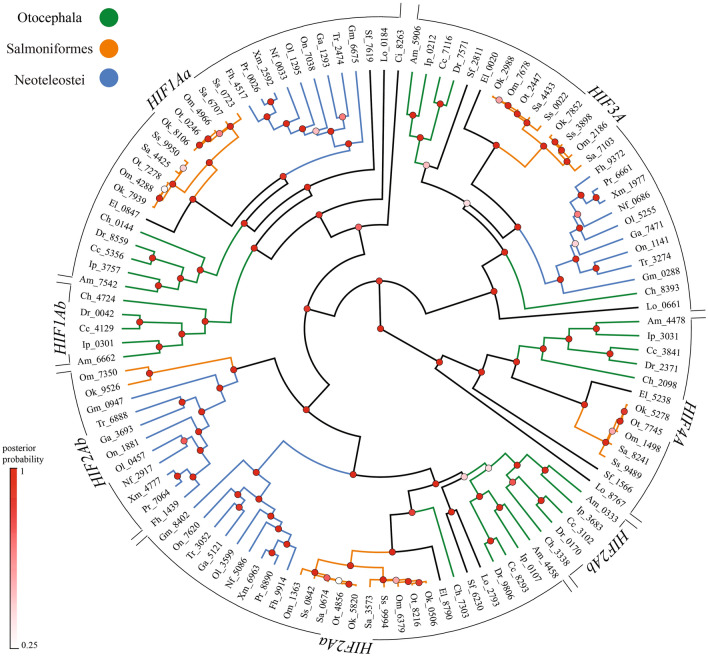
Phylogeny of actinopterygian *HIFA* coding sequences reconstructed by Bayesian inference. Analyses used the general time reversible model with six gamma categories and allowing for invariants (GTR+G+I). The tree with maximum clade credibility and mean heights is shown and the nodes are colored by posterior probability values. *HIFA* clades are shown on the circumference and the following taxa are color coded within each group: Otocephala, green; Salmoniformes, orange; Neoteleostei, blue. The outgroup, *Ciona intestinalis*, basal actinopterygian (spotted gar, *Lepisosteus oculatus*), basal teleost (Asian arowana, *Scleropages formosus*), and sister taxa to Salmoniformes (Northern pike, *Esox lucius*), are not color coded. Sequences are identified by the first letter of the genus and species followed by the last four digits of the NCBI or Ensemble reference gene accession number (see Supplemental Table S1 for a full list of genes).

A total of 114 putative *HIFA* homologs were recovered from searching the genomes of 22 species of Actinopterygii representing 14 orders (Supplemental Table S1). Phylogenetic analyses resolved four distinct clades (Fig. 1). This pattern was strongly supported by Bayesian analyses of nucleotide and deduced amino acid sequences (posterior probabilities ≥ 0.89; Supplemental Figs. S1, S3), as well as by maximum likelihood analyses (bootstrap values ≥ 0.71 for nucleotide analyses and ≥ 0.79 for amino acid analyses; Supplemental Figs. S2, S4). As expected, the branching patterns of species within each clade generally reflected the currently accepted phylogeny of fishes^37^. The spotted gar (*Lepisosteus oculatus*), a basal actinopterygian that diverged prior to the TGD, has one homolog in each clade. Thus, we infer that the four clades represent products of the two rounds of genome duplication in the ancestor of vertebrates and hereafter refer to these as *HIF1A*, *HIF2A, HIF3A,* and *HIF4A*. Most taxa examined here have at least one representative of all four *HIFA* genes, the exception being the most derived ray-finned fishes, the Neoteleostei, which appear to lack *HIF4A*.

### Teleost-specific *HIFA* paralogs

Teleost-specific duplicates of *HIF1A* (*HIF1Aa* and *HIF1Ab*) were only recovered in the Otocephala, a group including herrings, true minnows, carps, tetras, and catfish. Our results extend the observations of Rytkonen et al.^26^, who documented that the family Cyprinidae (e.g., zebrafish and carp) have teleost-specific paralogs of *HIF1A*, to include other Otocephala. Similarly, teleost-specific duplicates of *HIF2A* (*HIF2Aa* and *HIF2Ab*) are present in all the Otocephala examined here, as previously observed for cyprinids^26^. Additionally, more-derived fishes (Salmoniformes and their sister group, Esociformes, and Neoteleostei) retain a truncated version of *HIF2A*, previously referred to as a “relic” of the TGD^26^. The coding sequence of this truncated version is only one-third to one-half of “full-length” *HIF2A,* corresponding to the N-terminal portion of the protein (Supplemental Table S1). Although nucleotide and amino acid sequence analyses failed to reliably group it with the Otocephala *HIF2Ab*, evaluation of flanking genes placed the truncated form with other *HIF2Ab* (see below). Only one copy of *HIF3A* and *HIF4A* were recovered in any given species (with the exception of putative salmonid-specific paralogs, see below), which suggests that one duplicate of each of these genes was rapidly lost after the TGD. Previous analyses proposed that Cyprinidae retained teleost-specific duplicates of *HIF3A*^26^. Our analyses grouped one of these genes with *HIF3A* from spotted gar and one with *HIF4A* from spotted gar. Because spotted gar arose prior to the TGD, our results indicate that these cyprinid genes are *HIF3A* and *HIF4A*, rather than teleost-specific duplicates of *HIF3A*.

### Salmonid-specific *HIFA* paralogs

The current analysis revealed that Salmoniformes have two paralogs of *HIF1Aa*, *HIF2Aa*, and *HIF3A* (Fig. 1). These duplicates are not observed in the sister group Esociformes (Northern pike) and the branch lengths joining them are very short, consistent with an origin during the SGD. In support of this, Berthelot et al.^33^ noted that rainbow trout retained as many as 48% of the gene duplicates arising from the SGD, including an over-representation of transcription factors. In the absence of a naming convention for salmonid-specific duplicates, and to distinguish these from TGD duplicates, these paralogs are referred to as *HIF1Aa_s1*, *HIF1Aa_s2*, *HIF2Aa_s1*, *HIF2Aa_s2*, *HIF3A_s1*, and *HIF3A_s2*.

### Synteny analyses support relationships within paralogs

**Figure 2:**
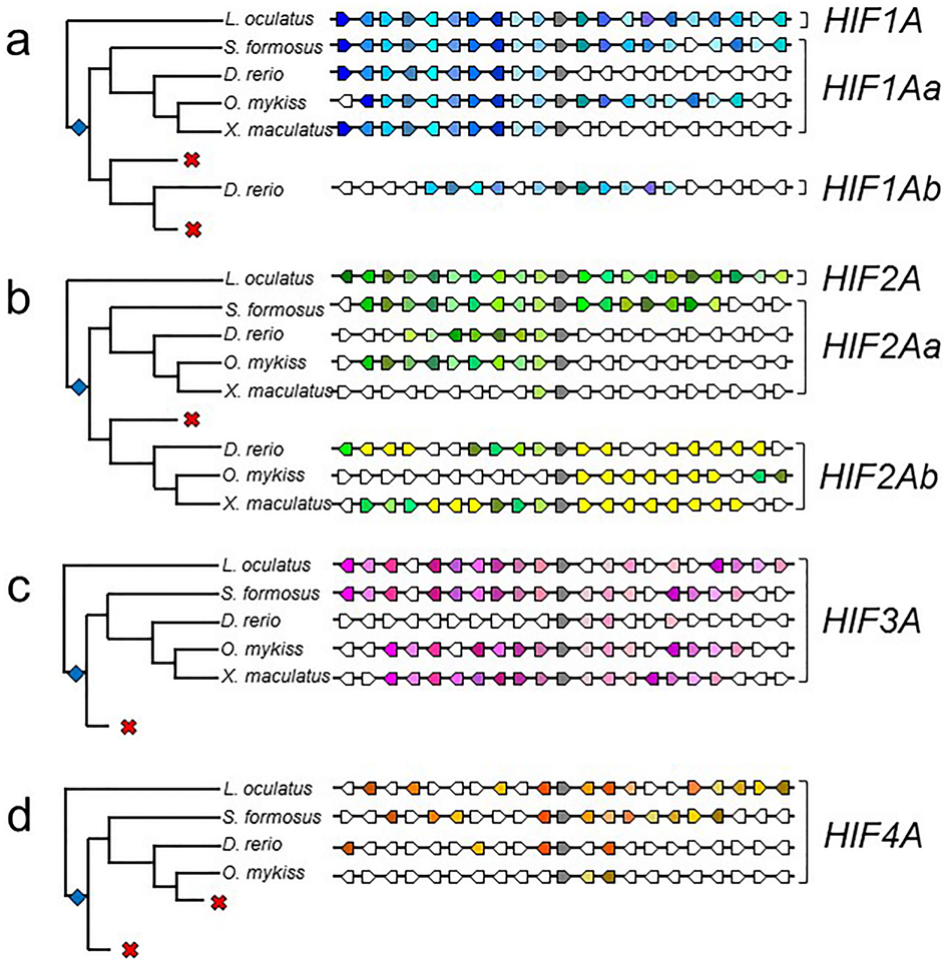
Synteny analysis of actinopterygian (**a**) *HIF1A*, (**b**) *HIF2A*, (**c**) *HIF3A*, and (**d**) *HIF4A*. The 10 flanking genes on either side of each *HIFA* paralog (gray arrows) are compared among spotted gar (*Lepisosteus oculatus*), Asian arowana (*Scleropages formosus*), zebrafish (*Danio rerio*), rainbow trout (*Onchorhynchus mykiss*), and southern platyfish (*Xiphophorus maculatus*). For each *HIFA* paralog, genes shared between spotted gar and more derived fishes are uniquely colored (see gene abbreviations in Supplemental Table S2). For *HIF2A*, genes shared among *HIF2Ab* but not present in *HIF2Aa* are shown in yellow. The relationships among fishes is from Hughes et al.^37^ and branch lengths do not indicate divergence times. The teleost-specific genome duplication is indicated by the blue diamonds, and putative losses of specific *HIFA* paralogs are shown by reds crosses.

Because the relationship of paralogs arising from the TGD based upon sequence analyses alone can be ambiguous^38^, we used shared synteny among species representing major fish lineages to clarify the relationships among *HIFA* paralogs (Fig. 2; Supplemental Table S2). For *HIF1A*, several flanking genes in spotted gar (*L. oculatus*) are conserved throughout Actinopterygii. As expected, there are more shared flanking genes in primitive species (*S. formosus*) compared to more derived species. Notably, the gene order of the 10 upstream genes is perfectly conserved in *HIF1Aa* in Otocephala (represented by *D. rerio*). This pattern supports the view that *HIF1Aa* of Otocephala is orthologous with the single paralog of *HIF1A* found in other ray-finned fishes. Although there are a similar number of flanking genes conserved between spotted gar and Otocephala *HIF1Ab*, their order and direction are more variable.

For *HIF2A*, the order of flanking genes is not as highly conserved across species, especially among Neoteleostei (represented by *X. maculatus*), where only the immediate upstream flanking gene is conserved in one of the two teleost-specific duplicates. This is the full-length form of *HIF2A* (i.e., not truncated) and its grouping with the full-length forms of *HIF2A* from other species is strongly supported by phylogenetic analyses (Fig. 1). Because this gene from all other species shares strong syntenic relationships with spotted gar *HIF2A*, these genes are *HIF2Aa*. The other teleost-specific paralog of *HIF2A* in Otocephala and Salmoniformes shares fewer flanking genes with spotted gar, and thus represents *HIF2Ab*, as previously proposed^26,39^. Importantly, up to 15 flanking genes are shared between *HIF2Ab* from zebrafish, *Danio rerio* (representing Otocephala), and the truncated *HIF2Ab* genes in Salmoniformes and Neoteleostei (Fig. 2, yellow arrows), a strong indication of common ancestry. Thus, we conclude that Otocephala *HIF2Ab* is orthologous with the truncated *HIF2Ab* in more derived species.

Synteny analysis of *HIF3A* and *HIF4A* showed considerable variation in the number of shared flanking genes among these paralogs in Actinopterygii. Interestingly, Otocephala *HIF3A* shares only three flanking genes with spotted gar, considerably fewer than observed in more-derived species. For *HIF4A*, the number of flanking genes roughly reflected the degree of divergence from the ancestral species, as expected, being highest in the Asian arowana (*S. formosus*) and the least in the rainbow trout (*O. mykiss*). The number of shared genes among *HIF1A*, *HIF2A*, *HIF3A*, and *HIF4A* was extremely limited (Supplemental Table S2), supporting an origin of these four paralogs in the ancient genome duplications at the base of vertebrate evolution.

Finally, synteny analysis of salmonid-specific duplicates of *HIF1Aa, HIF2Aa*, and *HIF3A* showed that one of the paralogs (s1) shares more flanking genes with the corresponding gene in Esociformes, the sister group of Salmoniformes, than the other paralog (s2) (Supplemental Table S2).

### Evidence for positive selection

We investigated whether *HIFA* genes experienced variable selective pressures using branch model tests performed in EasyCodeML^40^ on all accessions across four *HIFA* clades and one outgroup, *Ciona intestinalis*. A two-ratio model was not a better fit to the data than a one-ratio model in any of the tests, indicating that ω ratios (nonsynonymous to synonymous substitution ratios; dN/dS) were similar among all four *HIFA* genes (Table 2). The one-ratio model ω values were all much less than one, suggestive of overall purifying selection on each *HIFA* gene.

**Table 2:** Summary of EasyCodeML branch model analyses for detecting variable selective pressures among *HIFA* genes. The two-ratio Model 2 allowed the ratio of non-synonymous to synonymous substitutions (ω) in the indicated HIFA gene to differ from ω for other HIFA genes. Model 0 assumed a constant ω for all HIFA genes. For each HIFA gene, the difference in likelihood of the two models was evaluated with likelihood-ratio tests (LRT).

Foreground branch	Model	− lnL	Parameter estimates	LRT *P* value
*HIF1A*	Two ratio Model 2	932.93	ω0 = 0.0406, ω1 = 0.1139	0.463
	Model 0	933.20	ω = 0.0415	
*HIF2A*	Two ratio Model 2	933.20	ω0 = 0.0415, ω1 = 2.2060	0.994
	Model 0	933.20	ω = 0.0415	
*HIF3A*	Two ratio Model 2	933.30	ω0 = 0.0438, ω1 = 0.0260	0.378
	Model 0	932.91	ω = 0.0442	
*HIF4A*	Two ratio Model 2	932.50	ω0 = 0.0452, ω1 = 0.0001	0.121
	Model 0	933.71	ω = 0.0408	

**Figure 3:**
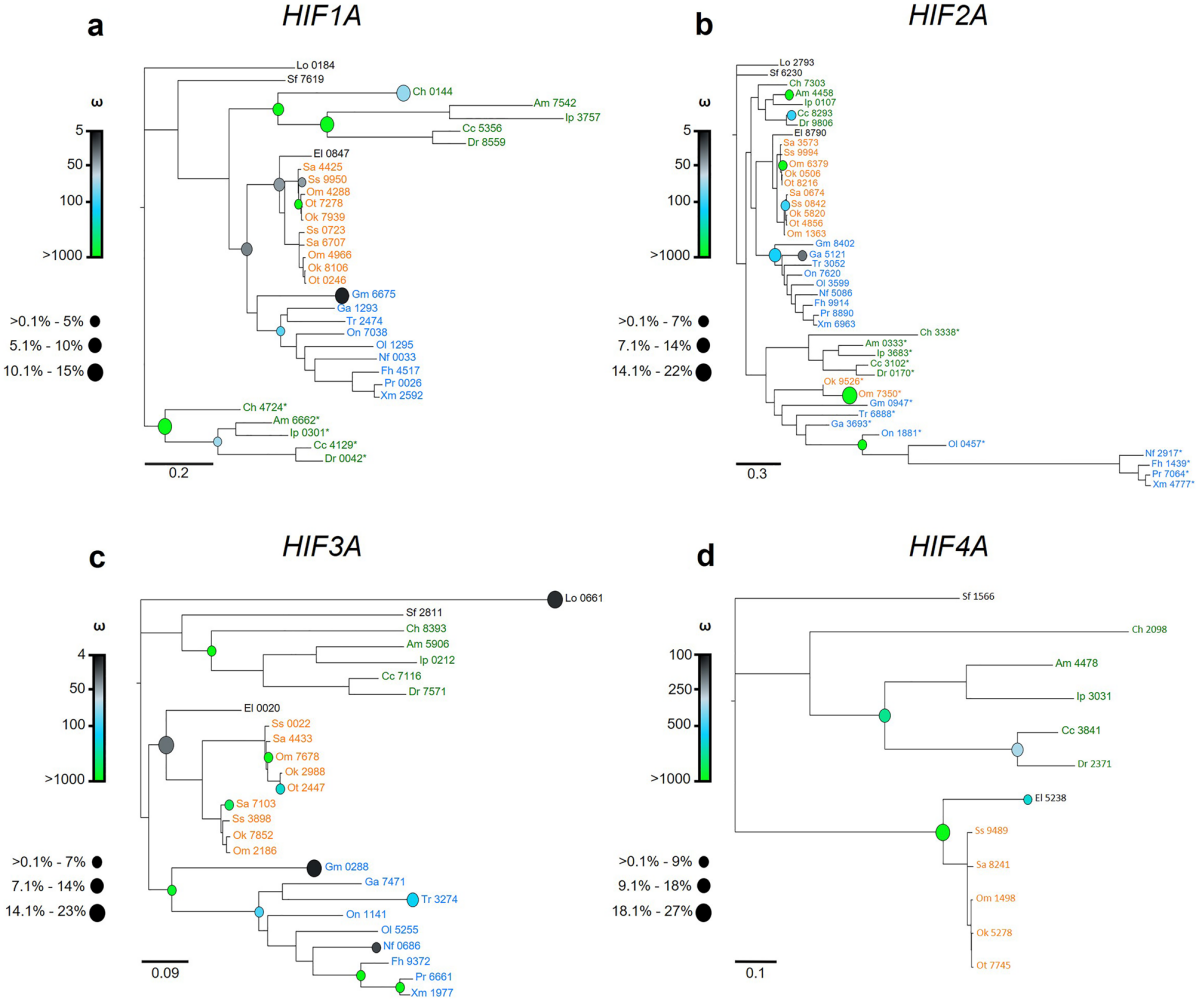
Positively selected branches in the evolution of actinopterygian (**a**) *HIF1A*, (**b**) *HIF2A*, (**c**) *HIF3A*, and (**d**) *HIF4A*. Branches were identified by adaptive branch-site random effects likelihood (aBSREL) tests for episodic diversification. The size of the circle represents the proportion of sites on branches significant for positive selection and the color gradient represents the magnitude of the positive selection ω rate class. For each *HIFA* paralog, taxa are color coded: Otocephala, green; Salmoniformes, orange; Neoteleostei, blue. The outgroup, *Ciona intestinalis*, basal actinopterygian (spotted gar, *Lepisosteus oculatus*), basal teleost (Asian arowana, *Scleropages formosus*), and sister taxa to Salmoniformes (Northern pike, *Esox lucius*), are not color coded. Sequences are identified by the first letter of the genus and species followed by the last four digits of the NCBI or Ensemble reference gene accession number (see Supplemental Table S1 for a full list of genes). Teleost-specific duplicates *HIF1Ab* and *HIF2Ab* are designated by asterisks.

Next, the four *HIFA* clades identified by phylogenetic analyses were independently examined for gene-wide and codon-based episodic and pervasive selection. Gene-wide tests of episodic selection performed with BUSTED^41^ found evidence of diversifying selection for at least one site on at least one branch of each *HIFA* gene (*HIF1A*: LRT = 46.754, *p* = 3.52e−11; *HIF2A*: LRT = 175.805, *p* = 0; *HIF3A*: LRT = 150.118, *p* = 0; *HIF4A*: LRT = 9.001, *p* = 0.006). This result was supported by aBSREL^42,43^ analyses showing evidence of diversifying selection for each *HIFA* (Fig. 3). The percentages of branches within each gene tree displaying significant positive selection were 18% for *HIF1A* (Fig. 3a), 10% for *HIF2A* (Fig. 3b), 19% for *HIF3A* (Fig. 3c), and 26% for *HIF4A* (Fig. 3d). For each gene, several of the branches that showed significant positive selection corresponded to major taxonomic groups. For example, significant positive selection was detected for the branch leading to Otocephala *HIF1Aa*, the branch leading to *HIF1Aa* in Salmoniformes and Neoteleostei, and the branch leading to Otocephala *HIF1Ab* (Fig. 3a).

Codons potentially under episodic or pervasive positive selection were detected for the four *HIFA* genes (Table 3). For all *HIFA* genes, episodic positive selection occurred more frequently than pervasive positive selection. MEME^44^ identified 25 sites under episodic positive selection for *HIF1A*, 41 sites for *HIF2A*, 34 sites for *HIF3A*, and 18 sites for *HIF4A*. Of this total of 118 sites, 60 also had BUSTED evidence ratios greater than two, providing further support that these sites have experienced episodic positive selection^41^. FEL^45^ showed that pervasive positive selection has acted on one site for *HIF1A*, two sites for *HIF2A*, two sites for *HIF3A*, and six sites for *HIF4A*. Two additional tests of pervasive selection, SLAC^45^ and FUBAR^46^, corroborated these results for one site in *HIF1A* and one site in *HIF2A*. Together, these analyses identified at least one site in each *HIFA* gene that has experienced both episodic and pervasive positive selection.

**Table 3:** Codons putatively under positive selection in *HIFA* genes in Actinopterygii.

Gene	Episodic Selection	Pervasive Selection
*HIF1A*	51^a^, 57^a^, 60^a^, 62^a^, 65, 140, 271^a^, 354, 365^a^, 484, 525, 579, 590, 595, 609^a^, 695, 761^a^, 774^a^, **835**, 841, 872, 875, 876^a^, 877, 931	**835**^b^
*HIF2A*	172, 204^a^, 210, 213, 219^a^, 222^a^, 224, 293, 304^a^, 306^a^, 357^a^, 371^a^, 373^a^, 380^a^, 382^a^, 385^a^, 386^a^, 388^a^, 389^a^, 392^a^, 394^a^, 396, 397^a^, 407, 425^a^, 452^a^, 577, 635^a^, 674^a^, 676, 694^a^, 746, 787^a^, 873, 921, 999^a^, **1063**, 1087, 1090, 1093^a^, 1095	94, **1063**^b,c^
*HIF3A*	26, 188^a^, 210^a^, 265^a^, 268^a^, 339, 436, **457**, 460, 464, 478^a^, 479^a^, 494, 529^a^, 590, 600^a^, 605^a^, 645, 661^a^, 675^a^, 713^a^, 772^a^, 773^a^, **774**, 775^a^, 788^a^, 792^a^, 802^a^, 807, 829^a^, 830^a^, 831^a^, 836^a^, 842^a^	**457**, **774**
*HIF4A*	68, 115, 124, 147, 287, 300, 362, **382**, 511, 530, 633, 666, 683, **703**, 735, 774, 783^a^, 796^a^	71, **382**, 654, 673, **703**, 727

Episodic positive selection was detected by MEME and pervasive positive selection was detected by FEL. Codons detected by both tests are shown in bold type. Codon number corresponds to the position in the multiple sequence alignment of all *HIFA* genes used in each analysis. See Supplemental Table S3 for amino acid identities in each species for each *HIFA* paralog.

^a^Episodic positive selection supported by BUSTED evidence ratio > 2.

^b^Pervasive positive selection supported by SLAC posterior probability > 0.95.

^c^Pervasive positive selection supported by FUBAR posterior probability > 0.95.

For each *HIFA* gene, the amino acid residues aligning with sites putatively under positive selection (Supplemental Table S3) were scored by their physicochemical properties^47^. Species were then grouped by discriminant analyses of principal components based upon these properties^48,49^. In agreement with the aBSREL analyses (Fig. 3), the physicochemical properties of these positively selected sites tended to group according to the species’ phylogenetic placement (Supplemental Fig. S5). In addition, these analyses distinguished the teleost-specific paralogs (*HIF1Aa/b* and *HIF2Aa/b*) from one another, with a few exceptions (e.g., *HIF1Ab* from the common carp, *Cyprinus carpio*, grouped with *HIF1Aa* from more-derived fishes). For *HIF3A* and *HIF4A*, the paralogs from Salmoniformes constituted a distinct group from the corresponding paralogs in other Actinopterygii. This analysis, however, did not discriminate between salmonid-specific paralogs for *HIF1Aa*, *HIF2Aa*, or *HIF3A*, likely reflecting their relatively recent origin.

### Structural modeling of HIFα amino acid variation

**Figure 4:**
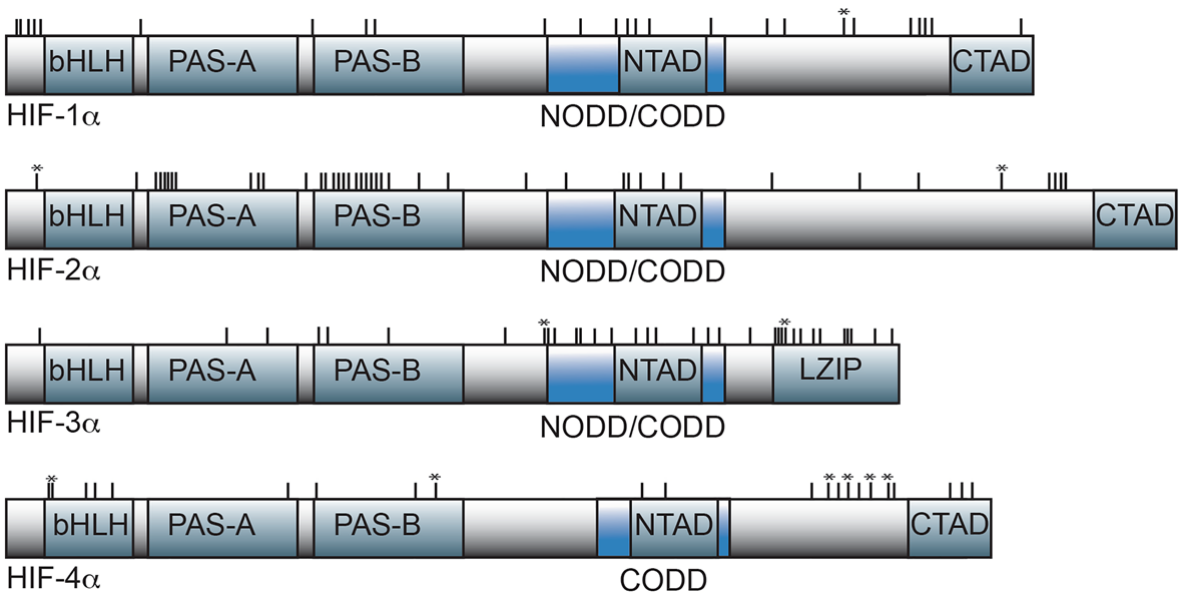
Location of amino acid sites under positive selection relative to the domain structure of HIFα subunits. Major structural domains are the basic-helix-loop-helix (bHLH), *PER*-*ARNT*-*SIM* (PAS-A, PAS-B), oxygen-dependent degradation (NODD and CODD), transactivation (NTAD and CTAD), and leucine zipper (LZIP) domains. The approximate locations of amino acid sites putatively under positive selection relative to these domains are shown with vertical hash marks above the domain models. The specific position and amino acid identity at each site in each HIFα subunit are given Supplemental Table S3. Asterisks indicate amino acid sites that are potentially under pervasive positive selection.

**Figure 5:**
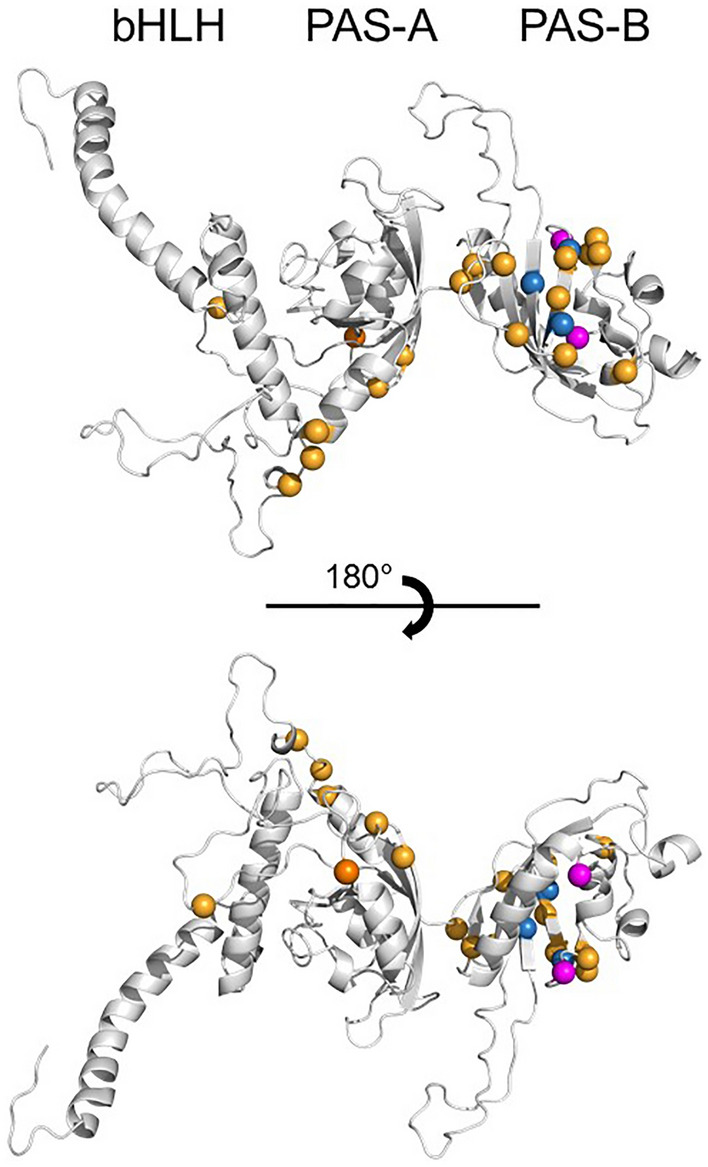
Structural model of the amino-terminal half of actinopterygian HIF2α showing sites putatively under positive selection. Twenty-three residues potentially under positive selection (MSA Codons 219–577, Table 3) mapped to a structural model of the N-terminal half of HIF2α based upon mouse HIF2α^50^. Two residues shown as purple spheres make contacts at the dimer interface between HIF2α and HIFβ and three residues shown as blue spheres contact an anti-cancer drug (0X3) known to interfere with subunit dimerization in mammals^50^. Eighteen other residues potentially under positive selection are shown as orange spheres. The upper and lower images are rotated by 180° around the indicated plane. Conserved DNA-binding (bHLH) and protein dimerization (PAS-A, PAS-B) domains are indicated at the top.

Sites potentially experiencing positive selection fell within protein domains responsible for DNA-binding (bHLH), protein dimerization (PAS-A and PAS-B), protein stability (NODD and CODD), or activation of target genes (NTAD and CTAD) for each HIFα subunit (Fig. 4). For HIF1α and HIF2α, we made structural models of their N-terminal halves based the corresponding regions from mammalian HIF1α and HIF2α^50^ and mapped the sites identified as being under positive selection for these two subunits. Only five of the 25 sites in HIF1α potentially under positive selection are found in this region of the protein (Fig. 4). Of these, one was the first amino acid of the bHLH domain, two fell in the PAS-B domain, and two were in loops connecting the major structural domains (Supplemental Fig. S6). For HIF2α, on the other hand, more than half of the 42 sites potentially under positive selection occur in the N-terminal half of the protein (Fig. 4), 23 of which mapped to a structural model of HIF2α (Fig. 5). The majority of these sites are in the PAS-A and PAS-B domains, and include five residues in the PAS-B domain that directly or indirectly interact with HIFβ (ARNT) in mammals^50^. Although structural models were not made for HIF3α or HIF4α, positively selected sites were found in the bHLH (HIF4α) or PAS domains (HIF3α and HIF4α) (Fig. 4). In addition, 13 of the 34 sites potentially under positive selection in HIF3α fell in the C-terminal leucine zipper (LZIP) domain specific to this HIFα subunit (Fig. 4). Collectively, these results suggest that amino acids involved in DNA-binding or protein dimerization may be under positive selection in Actinopterygii.

We next asked whether positive selection may have occurred at the N-terminal and C-terminal oxygen dependent degradation domains (NODD and CODD) that are potential targets of regulation by prolyl hydroxylases^8^ and the extreme C-terminal CEVN motif targeted by asparaginyl hydroxylase^51^. Across HIFα subunits, both the NODD and CODD were highly conserved, with a couple of notable exceptions. First, two sites aligning with alanine and proline in the canonical hydroxylation motif of LxxLAP in the NODD of HIF3α (MSA codons 463 and 464, Table 3) are putative targets of positive selection. Second, the NODD is absent in HIF4α. These observations support suggestions that the NODD may be less critical than the CODD in determining the oxygen-dependence of HIFα subunit degradation^8,52^. The current analysis also confirms that HIF3α lacks the asparaginyl hydroxylation motif, CEVN^21,53^. Moreover, the asparagine targeted by hydroxylation is absent in salmonid HIF4α, which have threonine at this position (Supplemental Table S3).

Although prolyl and asparaginyl hydroxylation are critical to the stability and transcriptional activity of HIFα subunits, respectively, the protein subunits are subject to a variety of other post-translational modifications (PTM) in mammals^54,55^. Accordingly, we determined if the sites potentially under positive selection in HIF1α and HIF2α from fishes aligned to sites of known PTM in humans. For HIF1α, one site (MSA codon 62) aligned with a lysine in humans (K11), which, when acetylated, blocks proteosomal degradation^54,56^. Four other sites (MSA codons 609, 695, 761, and 774) aligned with sites that are phosphorylated in human HeLa cells (S484, S581, S657, S664)^55^. At one of these sites (MSA codon 695), the residue in fishes is not phosphorylatable. For HIF2α, only one site (MSA codon 1093) aligned with a residue that is subject to phosphorylation in humans (S790). Finally, although not a site identified as under positive selection, it is relevant to note that the site that aligns with mammalian S31 is glycine in most fishes. In mammals, this residue is phosphorylated under hypoxia and may reduce the transcriptional activity of HIF1^55^. As documented by Daly et al.^55^, and substantiated here, only primitive fishes have a serine at this location, suggesting that this potential mechanism of transcriptional regulation has been lost in more-derived species of fish.

### Transcript analyses

**Figure 6:**
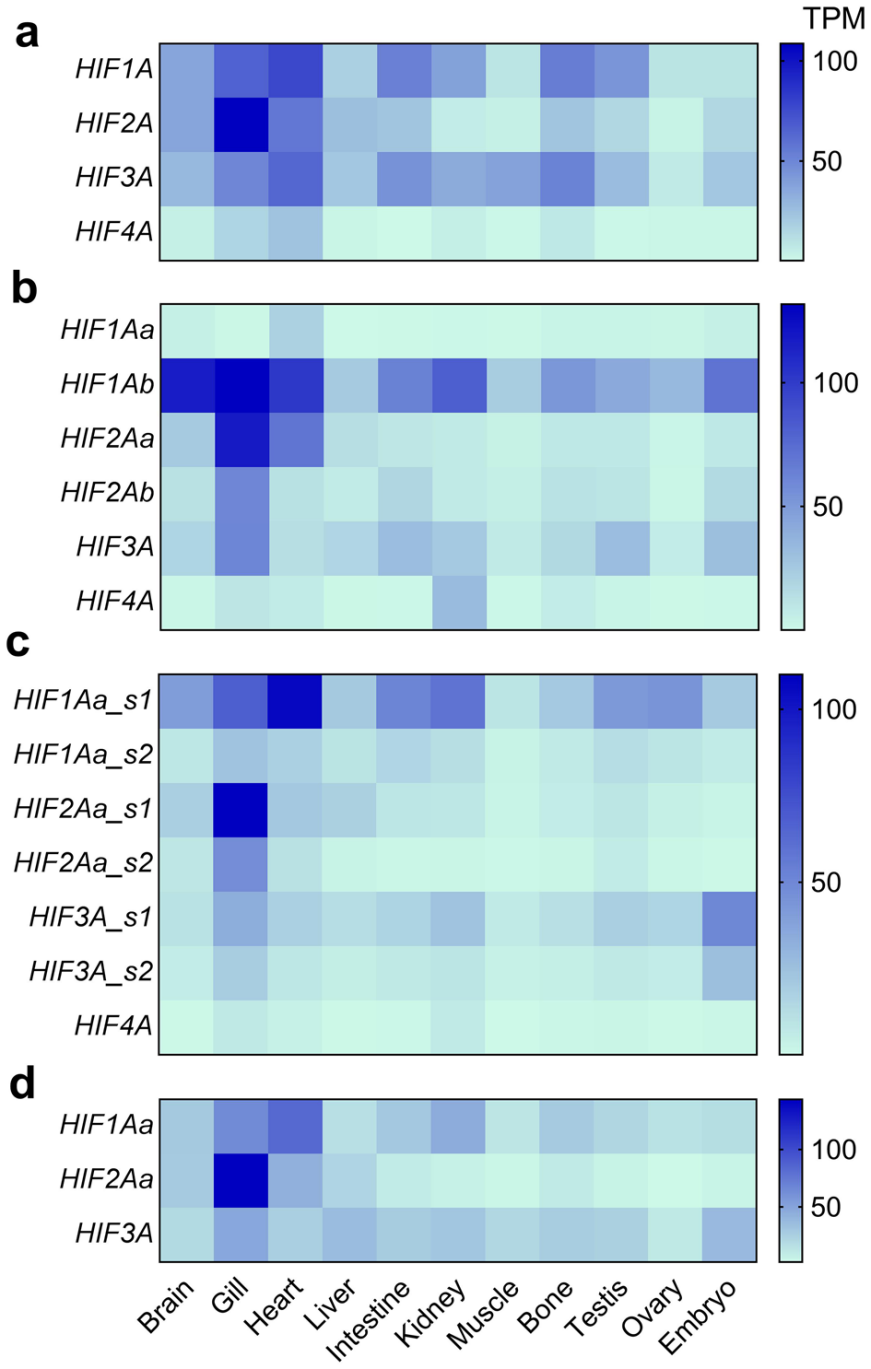
Expression of *HIFA* paralogs in tissues of Actinopterygii. (**a**) *HIFA* transcript abundance, in transcripts per million (TPM), in spotted gar (*Lepisosteus oculatus*) and other species expressing one form of each *HIFA*: silver arowana (*Osteoglossum bicirrhosum*), bowfin (*Amia calva*), European eel (*Anguilla anguilla*), Northern pike (*Esox lucius*), Eastern mudminnow (*Umbra pygmae*), (**b**) *HIFA* transcript abundance (in TPM) in Otocephala: Allis shad (*Alosa alosa*), zebrafish (*Danio rerio*), panga (*Pangasius hypophthalmus*), and Mexican tetra (*Astyanax mexicanus*); (**c**) *HIFA* transcript abundance (in TPM) in Salmoniformes: grayling (*Thymallus thymallus*), European whitefish (*Coregonus lavaretus*), brown trout (*Salmo trutta*), rainbow trout (*Onchorhynchus mykiss)*, and brook trout (*Salvelinus fontinalis*); (**d**) *HIFA* transcript abundance (in TPM) in species lacking *HIF4A*: Ayu sweetfish (*Plecoglossus altivelis*), Atlantic cod (*Gadus morhua*), medaka (*Oryzias latipes*), and European perch (*Perca fluviatilis*). The heatmap is based upon median TPM for a given paralog in each tissue (see Supplemental Table S4 for tissue-specific TPM values for each species).

The PhyloFish database^57^ was queried for *HIFA* transcripts in multiple tissues across a broad sampling of ray-finned fishes. In general, *HIF1A* demonstrated the broadest tissue distribution, being higher, on average, than the other *HIFA* paralogs in most tissues represented in the PhyloFish database (Fig. 6; Supplemental Table S4). Frequently, the highest levels of *HIF1A* transcripts were found in heart. *HIF2A* was more restricted in its distribution and, in many species, it was the most abundant paralog in gill. *HIF3A* was expressed at substantial levels in many tissues, being the most highly expressed paralog in embryo in several species. In those species having the *HIF4A* gene, its expression was low and limited to a few tissues (e.g., heart, gill, kidney, and bone).

The Otocephala are the only lineage of ray-finned fishes to retain both teleost-specific paralogs of *HIF1A* (see above). While other lineages exclusively express *HIF1Aa*, Otocephala express *HIF1Ab* more highly and broadly across tissues, with only very low expression of *HIF1Aa* (Fig. 6b). On the other hand, Otocephala are like other fishes in expressing more *HIF2Aa* than *HIF2Ab*, especially in gill. In Otocephala, *HIF2Ab* encodes a “full-length” protein and it was also expressed in gill. Among the Salmoniformes, which have salmonid-specific duplicates of *HIF1Aa*, *HIF2Aa*, and *HIF3A* in their genomes, one paralog of each was preferentially expressed (Fig. 6c). *HIF1Aa_s1* was broadly distributed and most abundant in heart; *HIF2Aa_s1* was largely restricted to gill; low levels of *HIF3A_s1* were detected in many tissues and was it the most abundant paralog in embryo. In each case, the expression of the other paralog (s2) was lower and showed similar tissue distribution.

*HIFA* expression in more derived species reflected broad tissue distribution of *HIF1Aa* and *HIF3A*, with more restricted, gill-specific expression of *HIF2Aa*, as seen in other species (Fig. 6d). The truncated form of *HIF2Ab*, which is present in the genomes of several fish lineages (see above), was not recovered in the PhyloFish database. The transcript for *HIF4A* was not found in any Neoteleostei represented in the PhyloFish database, consistent with its absence from genomes of more-derived Actinopterygii.

## Discussion

### Genome duplication and *HIFA* diversity among ray-finned fishes

Two rounds of genome duplication in the ancestor of vertebrates, followed by additional genome duplication during the evolution of ray-finned fishes, expanded certain gene families, including those encoding HIF, a master regulator of oxygen-dependent gene expression in animals. The present analyses of *HIFA* genes in Actinopterygii revealed that several lineages retain four paralogs predicted from two rounds of genome duplication at the base of vertebrate evolution. The current results suggest that several sequences formerly described as “*HIFA-like*” or “*HIF1A-like*”^32^ should be recognized as either *HIF3A* or *HIF4A*. Although *HIF3A* has been previously described in vertebrates, including fish^21,26,58^, there has been no formal recognition of *HIF4A* in any vertebrate animal. Rytkonen et al.^26^ presented evidence that certain fishes possess *HIF3Ab*, a putative teleost-specific duplicate of *HIF3A*. We show that this gene occurs in the genome of spotted gar, representing a lineage of ray-finned fishes that diverged prior to the TGD. Thus, it is properly designated as *HIF4A*. This gene is found in all ray-finned fishes examined here with the exception of the more-derived Neoteleostei. Thus, *HIF4A*, a heretofore “missing Ohnolog”, is widely, but not uniformly, distributed among Actinopterygii.

Consistent with Rytkonen et al.^26^, we found teleost-specific paralogs of *HIF1A* and *HIF2A* in several lineages. For *HIF1A*, we present evidence that all Otocephala, not just the Cyprinidae, retain both paralogs. Similarly, duplicated “full-length” forms of *HIF2A* are present in all Otocephala examined here. This is significant because some species in the Otocephala are not particularly tolerant of low oxygen, meaning that retention of teleost-specific duplicates does not necessarily confer hypoxia tolerance. Rather, having duplicated *HIFA* genes could have permitted the evolution of hypoxia tolerance in certain lineages (e.g., cyprinids) given the proper ecological context (e.g., persistent or recurrent aquatic hypoxia)^31^. In addition, we found that a truncated form of *HIF2Ab* is more broadly distributed among ray-finned fishes than previously appreciated^26^. Only a single *HIF3A* and *HIF4A* were recovered in the species examined here, however, arguing that one teleost-specific duplicate was quickly lost after the TGD, consistent with the notion that nonfunctionalization is the most common fate of one paralog after gene duplication^30^. We also report, for the first time, the presence of duplicates of *HIF1Aa*, *HIF2Aa*, and *HIF3A* in Salmoniformes, which likely arose from the SGD.

### A phylogenetically-based gene nomenclature

The duplication of *HIFA* genes during the TGD followed by the subsequent lineage-specific loss of various paralogs has given rise to an inconsistent nomenclature. Herein, we adopt two naming conventions that recognize the evolutionary relationships of teleost-specific paralogs^59^. First, when one or more lineage retains both paralogs, the “a” form is the one that shares more flanking genes with the ancestral (gar) form. Applying this rule to *HIF1A* results in *HIF1Aa* and *HIF1Ab* that conform to the gene names currently recognized. For *HIF2A*, however, conclusions based upon synteny differ from the names of *HIF2A* paralogs in some fishes, most notably zebrafish, *Danio rerio*. Here, we show that in most species, including zebrafish, the paralog we propose as *HIF2Aa* shares more syntenic genes with gar *HIF2A* than the other paralog. Moreover, this paralog was the first *HIF2A* described in fishes^60^ and, when teleost-specific paralogs were initially described, it was referred to as *HIF2Aa*^26^. The paralog we propose as *HIF2Ab* shares fewer flanking genes with gar, is less broadly expressed among tissues and species, and is predicted to encode a truncated protein in fishes other than Otocephala^26,39^. In zebrafish, the “a” and “b” designations are reversed. That is, the paralog we suggest is properly designated as *HIF2Aa* is *hif2ab* (or *epas1b*) in zebrafish (located on chromosome 13) and the paralog we propose as *HIF2Ab* is *hif2aa* (or *epas1a*) in zebrafish (located on chromosome 12). This discrepancy arises because in zebrafish, “the a or b suffix does not indicate primacy of publication and will be assigned purely based on the suffix of the surrounding genes” (https://zfin.atlassian.net/wiki/spaces/general/overview). Gasanov et al.^59^ suggest that this convention lacks phylogenetic context and should be revisited as the syntenic relationships between individual paralogs and ancestral fishes are elucidated, as we have now done for *HIF2A*. Until there is consensus, however, great care will be needed when interpreting reports of paralog-specific differences in *HIF2A*.

The second naming convention applies when no lineage retains both paralogs, as observed for *HIF3A* and *HIF4A* in the current study. In these cases, the relationship of the paralog that has been retained in different lineages is not certain. Although one might expect that all extant species retained the same teleost-specific duplicate, it is possible that one lineage retained one duplicate and another lineage retained the other copy (i.e., reciprocal silencing^30^). Indeed, the very limited shared gene order between Otocephala *HIF3A* and *HIF3A* in other ray-finned fishes suggests that might have occurred for this gene. Without clear evidence of the relationships of the paralogs to the ancestral form, the use of “a” and “b” should be avoided^59^.

Finally, the same conventions can be applied to paralogs arising during other gene duplication events, for example the SGD. Here, we propose that duplicates of *HIF1Aa*, *HIF2Aa*, and *HIF3A* that share more flanking genes with the sister group, Esociformes, be recognized as the “s1” paralog and the duplicate that shares fewer flanking genes be the “s2” paralog.

### Potential causes and functional consequences of *HIFA* diversity

In our comparison of the strength of natural selection acting on *HIFA* genes, we found all four clades had very low rates of nonsynonymous to synonymous substitutions (dN/dS; ω), suggesting that *HIFA* is subject to purifying selection. This result is consistent with natural selection acting to conserve the sequences of critical regulatory proteins, including transcription factors, and it agrees with previous studies reporting low values of ω for *HIFA* genes in fishes^16,32,61–63^. In addition, we found that values of ω were not statistically different when comparing gene clades to one another (i.e., *HIF1A, HIF2A, HIF3A*, and *HIF4A*). This result differs from that of Rytkonen et al.^16^, who reported that, among fishes, ω was equivalent for *HIF1A* and *HIF2A* and slightly, but significantly, lower than that for *HIF3A*. Based upon this, Rytkonen et al.^16^ proposed that *HIF3A* was evolving under relaxed purifying selection or adaptive positive selection. Our study differs from Rytkonen et al.^16^ in many ways, including the number and specific sequences used, the species designated as outgroup, and our grouping of some sequences formerly classified as *HIF3A* as *HIF4A* (see above). Despite these differences, the studies are similar in the conclusion that a major theme in *HIFA* evolution is one of purifying selection.

Against this backdrop of purifying selection, however, we found evidence of widespread episodic positive selection when each *HIFA* clade was independently evaluated with gene-wide and codon-based tests of positive selection. This finding is consistent with the idea that natural selection is episodic, but the strength of this signal may be overshadowed by strong purifying selection acting on other branches^44^. Our findings are in general agreement with studies on cyprinid fishes showing positive selection acting on specific genes or lineages^23,26,62,64^. Although we did not formally test whether teleost-specific paralogs are experiencing differing rates of selection (cf.^26^), branches leading to *HIF1Aa* and *HIF1Ab* were characterized by having a high proportion of sites under significant positive selection. Grouping *HIFA* genes according to the physicochemical properties of their deduced amino acid sequences provided further evidence of divergence between teleost-specific paralogs of *HIF1A* and *HIF2A*. For *HIF3A* and *HIF4A*, gene-wide and codon-based tests showed significant positive selection in branches leading to major taxonomic groups (e.g., Salmoniformes), which was likewise supported by divergence in the physicochemical properties of the translated proteins.

When the sites putatively under positive selection were mapped to the respective subunits’ sequences, several fell within conserved protein domains. Consistent with studies in fishes and vertebrates in general, several sites potentially under positive selection in HIFα subunits occur in the PAS domains, which are involved in DNA binding and subunit dimerization^15,16,32,64^. Pamenter et al.^15^ reviewed the amino acid sites diverging in high-altitude species or populations of terrestrial vertebrates, mainly mammals. Similar to the results reported here, they found more divergent amino acid sites in HIF2α than in HIF1α, with a preponderance of those sites in the PAS domains. Amino acid variation in the PAS domains is speculated to affect dimerization with HIF1β (ARNT), post-translational modification, and transcriptional activation^15^. Intriguingly, sites potentially under positive selection in *HIF2A* resolved in the current study mapped to two amino acids that contact HIF1β in mammals and three other sites that bind to compounds interfering with subunit dimerization^50^. Other sites in ray-finned fish *HIFA* genes that appear to be under positive selection mapped to amino acids that are subject to post-translational modification in human HIF1α or HIF2α, alter the sequence of a canonical prolyl hydroxylation domain in HIF3α, or mutate the target of asparaginyl hydroxylation in HIF4α. Whether the amino acid variation we report here affects HIFα protein stability or function, as reported for mammalian HIFα subunits^15^, remains largely unexplored in fishes.

### Tissue expression of *HIFA* in actinopterygii

Our survey of *HIFA* expression across Actinopterygii supports the idea that *HIF1A* is broadly expressed across tissues and that *HIF2A* is more restricted in its distribution. Interestingly, the tissue showing highest *HIF2A* expression levels is gill. While elevated levels of *HIF2A* in gill have been documented in single-species studies^39,65,66^, our results show that this pattern is broadly distributed among ray-finned fishes. Recently, Pan et al.^67^ showed that *HIF2A* is highly expressed in neuroepithelial cells of zebrafish gill, suggesting it might play a role in oxygen sensing by this tissue, analogous to its role in mammalian carotid body^68^. In addition, gills are highly vascularized, and the presence of *HIF2A* transcripts could reflect a large proportion of endothelial cells, which are known to express *HIF2A* in mammals. The current results also demonstrate that *HIF3A* is expressed at substantial levels in several tissues, being the most highly expressed *HIFA* in embryos in many species. Previous studies have shown that *HIF3A* is broadly expressed among fish tissues including embryos^39,52,58,66,69^. In zebrafish embryos, Kopp et al.^69^ documented an increase in *HIF3A* transcripts during exercise, and Zhang et al.^52^ demonstrated that *HIF3A* acts as a hypoxia-dependent transcriptional activator during early zebrafish development. Across all Actinopterygii, *HIF4A* was the least expressed *HIFA* transcript. In general, the level of *HIF4A* declined from primitive to more-derived species, being lost from the genome and, consequently, not expressed in Neoteleostei. This pattern is consistent with nonfunctionalization of *HIF4A* during the evolution of ray-finned fishes. Of note, *HIF4A* is also missing from the genomes of other vertebrates, suggesting it has been nonfunctionalized in these lineages as well^32^.

The current survey of *HIFA* expression highlights processes that may serve to maintain paralogs arising from the TGD. It has been argued that subfunctionalization has been an important force in maintaining both teleost-specific duplicates of *HIF1A* and *HIF2A* in zebrafish, a member of the Otocephala^26^. In the case of *HIF1A*, one striking result is that *HIF1Ab* is highly expressed across a broad array of tissues in Otocephala, a pattern displayed by the other paralog, *HIF1Aa,* in other Actinopterygii. In Otocephala, this might have allowed *HIF1Aa* to assume a different role, for example during development^26^; such subdivision of functions was not possible in other Actinopterygii that lost *HIF1Ab*. In Otocephala, levels of *HIF2Aa* transcripts were higher than *HIF2Ab,* as previously reported for zebrafish^66^, although still limited in its tissue distribution (see above). Although levels of *HIF2Ab* transcripts were quite low in Otocephala, they are reported to respond robustly to low oxygen exposure, at least in zebrafish^26^. The truncated transcript predicted from *HIF2Ab* in other Actinopterygii was not recovered from the PhyloFish database, but it has been found in transcriptomic studies, albeit at very low levels^39,70^. Because tissues used to generate the RNA for the PhyloFish database were from fish held under standard laboratory conditions, we cannot rule out the possibility that the expression of “truncated” *HIF2Ab* increases under other conditions (e.g., hypoxia) or at different developmental stages.

For duplicated forms of *HIFA* arising from the SGD, one paralog of *HIF1Aa*, *HIF2Aa*, and *HIF3A* was more highly expressed than the other across all tissues. The observation that the other paralog was expressed in the same tissues, albeit at lower levels of expression, suggests that differing tissue specificity does not account for the maintenance of both duplicates. As mentioned above, RNA was derived from a limited number of individuals sampled under relatively benign conditions, and the less-expressed paralog may be upregulated during different environmental conditions or developmental stages. But, because salmonids generally occur in well-oxygenated habitats and have poor hypoxia tolerance, there does not appear to be a link between *HIFA* duplication and hypoxia tolerance in this group. This suggestion is supported by the observation that Northern pike, from the sister group to Salmoniformes that diverged prior to the SGD, lacks the salmonid-specific duplicate but are more hypoxia tolerant than salmonids^71^. It is possible that these duplicates may play other roles in salmonid physiology or life-history, and future research is needed to evaluate whether subfunctionalization or neofunctionalization are playing a role in maintaining these salmonid-specific duplicates. Alternatively, they may be destined for nonfunctionalization, a process that is likely still underway in this lineage given the recency of the SGD^33^.

## Conclusions

Here, we demonstrate that the diversity of *HIFA* genes in Actinopterygii is greater than previously appreciated, provide evidence that episodic positive selection is involved in generating this diversity, and report paralog- and tissue-specific *HIFA* expression levels. The current results present several opportunities for future research on *HIFA* in fishes. For example, tolerance to hypoxia measured at the organismal level demonstrates a strong phylogenetic signal among ray-finned fishes^72^, and future research could assess whether hypoxia-tolerant lineages are associated with the specific amino acid variants in HIFα subunits reported here. Furthermore, in fishes as in other vertebrates, *HIF1A* has received considerably more attention than the other *HIFA* paralogs. The tissue expression of *HIF2A* and *HIF3A* suggest that these paralogs may play critical roles in specific tissues, gill and embryo, respectively, that warrant further study. We also document that a truncated form of *HIF2Ab* is widespread in the genomes of ray-finned fishes. If transcribed and translated, the predicted protein product would have characteristics that could allow it to negatively regulate oxygen-dependent gene expression, as demonstrated for splice variants of mammalian *HIF3A*^19,21^. Finally, there is increasing appreciation of the hypoxia-independent roles of HIF signaling^23,25^. Perhaps some of the diversity in *HIFA* among ray-finned fishes is explained by functions other than regulation of oxygen-dependent gene expression. The current genomic and transcriptomic analyses may serve as a roadmap for the continued study into HIF signaling during normal fish development and physiology, as well as in the response of fishes to increasingly challenging environments.

## Methods

### Data

All Actinopterygian genomes available at NCBI (https://www.ncbi.nlm.nih.gov) or Ensemble (http://www.ensembl.org/) through June 2020 were searched for *HIFA* genes using known or putative *HIF1A*, *HIF2A*, *HIF3A,* and *HIFA*-like transcript sequences. The corresponding coding sequences (CDS) were checked to ensure each was complete and, when multiple CDSs were available for a single locus, the longest sequence was retained. This resulted in a list of 122 sequences from 24 species. Sequences for two species (*Cynoglossus semilaevis* and *Maylandia zebra*) were not included because they were less well annotated and did not substantially contribute to the taxonomic breadth represented by the other eight Neoteleostei. The final sequence list included 114 sequences from 22 actinopterygian species plus one sequence from the sea squirt, *Ciona intestinalis*, as an outgoup (see Supplemental Table S1). Three-dimensional protein structural models were based upon mouse HIF1α-ARNT-DNA (4ZPR) and HIF2α-ARNT-DNA (4ZPK) crystal structures from the Protein Data Bank (http://www.wwpdb.org/)^50^. Data for the analyses of *HIFA* transcript abundance were obtained from the PhyloFish database (http://phylofish.sigenae.org/index.html)^57^.

### Phylogenetic and synteny analyses

Multiple sequence alignments (MSA) were made using MAFFT version 7.123b^73,74^ implemented through the GUIDANCE2 server with Max-iterate of 20 (http://guidance.tau.ac.il/ver2/). For phylogenetic analyses, we used the MSA with the default column cutoff of below 0.93^75,76^. Bayesian analyses were performed in BEAST 2 v2.6.1 (https://www.beast2.org/) applying an uncorrelated log-normal relaxed molecular clock model^77^, a Yule model prior, with 10,000,000 chain-length and 100,000 burn-in^78,79^. The best-fit model of nucleotide substitution was identified as the GTR+I+G model by both jModelTest2^80,81^ and ModelTest-NG^82,83^. Therefore, nucleotide analyses employed a general time reversible codon substitution model allowing for invariants and six gamma categories (GTR+I+G). Analyses of amino acid sequences employed a JTT matrix-based model^84^ allowing for invariants and six gamma categories, following a recent analysis of metazoan HIF-family proteins^32^. The maximum clade credibility tree was selected using TreeAnnotator v2.6.0^85^. Maximum likelihood analyses were performed using MEGAX v10.1.8^86^ with 100 bootstrap replicates^87^ under the same conditions used in Bayesian analyses. The trees with the highest log-likelihood were visualized and edited in FigTree v1.4.4 (http://tree.bio.ed.ac.uk/software/figtree/).

Shared synteny among representative species was assessed by determining the 10 deduced open reading frames (ORF) upstream and downstream of each putative *HIFA* gene using the NCBI Graphical Sequence Viewer (v3.38.0). If a putative ORF lacked a clear identification, BLASTP was used to compare the deduced protein sequence against Actinopterygii. For genes lacking an abbreviation at NCBI, the gene name was used in a search of UniprotKB (https://www.uniprot.org), and the corresponding abbreviation was used. A small number of putative ORFs could not be identified and were kept in the analysis as “unknowns”.

### Selection analyses

Translation alignments of full-length *HIFA* nucleotide sequences for selection analyses were created in Geneious v11.1.5 (https://www.geneious.com) using Clustal W^88^ alignment and BLOSUM^89^ substitution matrix. For each data set, we inferred the maximum likelihood gene tree using rapid hill-climbing mode in RAxML v8.2.0^90^ as implemented through the CIPRES Science Gateway^91^. This was accomplished by drawing bipartition information on the best tree from 100 trees using the GTRGAMMA substitution model based on 1000 non-parametric bootstrap replicates. We replaced characters for frameshifts and stop codons, as required for selection analyses, with the exportAlignment program in MACSE v2.00^92^. Evolutionary selection analyses were conducted using branch models in EasyCodeML^40^ to explore differences in dN/dS (ω) ratios among *HIFA* gene clades. Four branch models were performed independently by selecting a particular *HIFA* gene clade as the foreground (e.g., *HIF1A*) and remaining clades as the background. Nested models were compared using likelihood-ratio tests (LRT)^93^ to assess significance of log-likelihood ratios between a one‐ratio model (Model 0) that assumes a constant ω throughout the tree and a two‐ratio model (Two-ratio Model 2) that allows ω for foreground branches to differ from branches throughout the rest of the tree^94^.

Additional tests of gene-wide and codon-based episodic (at a subset of sites or branches) and pervasive (across the whole phylogeny) selection were performed for individual *HIFA* gene subsets in the HyPhy package^95,96^ through the Datamonkey webserver^97–99^. To assess whether a gene has experienced positive (diversifying) selection at any site on at least one branch given a phylogeny, we implemented the Branch-site Unrestricted Statistical Test for Episodic Diversification (BUSTED)^41^. To test whether episodic selection occurred on any branch at a subset of sites in a gene, we used adaptive Branch-Site Random Effects Likelihood (aBSREL)^42,43^. We also assessed whether individual sites were subject to episodic selection on a proportion of branches using a Mixed Effects Model of Evolution (MEME)^44^, and pervasive selection with Fixed Effects Likelihood (FEL)^45^, Fast Unconstrained Bayesian AppRoximation (FUBAR)^46^, and Single-Likelihood Ancestor Counting (SLAC)^45^.

For each *HIFA* clade, we compiled an X-matrix of the amino acids at the sites identified as being putatively under positive selection by the HyPhy selection analyses. The physicochemical properties of each amino acid were scored by five z-descriptors as described by Sandberg et al.^47^: z1 (hydrophobicity), z2 (steric bulk), z3 (polarity), z4, and z5 (the latter two both related to electronic effects). We used the adegenet package^48,49^ in RStudio^100^ to identify the number of clusters across species by applying the *k*-means algorithm, then performed discriminant analysis of principal components on the minimum number of retained principal components.

### Protein structural modeling

Three-dimensional protein models for actinopterygian HIF1α and HIF2α were derived by structural homology modeling based upon the HIF1α:ARNT:DNA and HIF2α:ARNT:DNA complexes from mouse^50^. These structures correspond to residues 13-357 of mouse HIF1α (GenBank AAH26139.1) and residues 3-361 of mouse HIF2α (GenBank AAH57870.1), respectively. Three-dimensional models were built with Modeller v10.1, using align2d and the standard single-template "automodel” modeling protocol^101^. For both HIF1α and HIF2α, five models were produced, and the models with the lowest molpdf and DOPE scores were chosen as representative for further study. The amino acid sites putatively under positive selection in Actinopterygii were mapped to these structures using PyMol (v2.5.1).

The N- and C-terminal oxygen-dependent degradation domains (NODD and CODD) and the C-terminal asparaginyl hydroxylation motif (CEVN) were identified from multiple sequence alignments in Jalview v2.10.5^102^. The NODD and CODD included the canonical LxxLAP sequence targeted by prolyl hydroxylases and adjacent residues known to play a role in oxygen-dependent regulation of HIFα^103^.

### *HIFA* transcript analyses

The PhyloFish database contains RNA-seq raw counts from multiple tissues for 23 species representing all major lineages of ray-finned fishes^57^. The tissues represented are brain, liver, gill, heart, skeletal muscle, kidney, bones, intestine, ovary (derived from a single female), testis (derived from a single male), and embryos. Prior to tissue sampling, fish were maintained under standard laboratory conditions (i.e., adequate aeration). Other details of library construction, sequencing, and quality control are found in Pasquier et al.^57^.

Data were downloaded for 19 species: spotted gar (*Lepisosteus oculatus*), silver arowana (*Osteoglossum bicirrhosum*), bowfin (*Amia calva*), European eel (*Anguilla anguilla*), Allis shad (*Alosa alosa*), zebrafish (*Danio rerio*), panga (*Pangasius hypophthalmus*), Mexican tetra (*Astyanax mexicanus*), Northern pike (*Esox lucius*), Eastern mudminnow (*Umbra pygmae*), grayling (*Thymallus thymallus*), European whitefish (*Coregonus lavaretus*), brown trout (*Salmo trutta*), rainbow trout (*Onchorhynchus mykiss*), brook trout (*Salvelinus fontinalis*), Ayu sweetfish (*Plecoglossus altivelis*), Atlantic cod (*Gadus morhua*), medaka (*Oryzias latipes*), and European perch (*Perca fluviatilis*). BLASTn with sequences for each *HIFA* paralog (Supplemental Table S1) were used to find *HIFA* transcripts for each tissue in each species. Transcripts were normalized for gene length and total reads by determining reads per kilobase per million transcripts (RPKM). For each *HIFA* in each tissue, transcripts per million (TPM) were calculated as $10^{6} {*}\frac{{{\text{RPKM}}}}{{\left( {{\text{sum}}\,{\text{RPKM}}} \right)}}$^104^. Results are presented as heatmaps of median TPM values for each paralog for each tissue (GraphPad Prism v8.0.0; San Diego, California, USA).

### Ethics approval

This study did not use animal or human subjects.

## Supplementary Information

Supplementary Table S1.

Supplementary Table S2.

Supplementary Table S3.

Supplementary Table S4.

Supplementary Figures.

## Data Availability

The data underlying this article were downloaded from the National Center for Biotechnology Information (https://www.ncbi.nlm.nih.gov/) or Ensembl (https://ensembl.org/index.html). Nucleotide and amino acid multiple sequence alignment files have been deposited at https://doi.org/10.6084/m9.figshare.21713759.v1^105^.

## References

[1] Breitburg D (2018). Declining oxygen in the global ocean and coastal waters. Science.

[2] Deutsch C, Ferrel A, Seibel B, Portner H-O, Huey RB (2015). Climate change tightens a metabolic constraint on marine habitats. Science.

[3] Deutsch C, Penn JL, Seibel B (2020). Metabolic trait diversity shapes marine biogeography. Nature.

[4] Kaelin WG, Ratcliffe PJ (2008). Oxygen sensing by metazoans: The central role of the HIF hydroxylase pathway. Mol. Cell.

[5] Semenza GL (2011). Oxygen sensing, homeostasis, and disease. N. Engl. J. Med..

[6] Gu YZ, Hogenesch JB, Bradfield CA (2000). The PAS superfamily: Sensors of environmental and developmental signals. Annu. Rev. Pharmacol. Toxicol..

[7] McIntosh BE, Hogenesch JB, Bradfield CA (2010). Mammalian Per-Arnt-Sim proteins in environmental adaptation. Annu. Rev. Physiol..

[8] Epstein AC (2001). *C. elegans* EGL-9 and mammalian homologs define a family of dioxygenases that regulate HIF by prolyl hydroxylation. Cell.

[9] Ivan M (2001). HIFα targeted for VHL-mediated destruction by proline hydroxylation: Implications for O2 sensing. Science.

[10] Jaakkola P (2001). Targeting of HIF-alpha to the von Hippel–Lindau ubiquitylation complex by O2-regulated prolyl hydroxylation. Science.

[11] Wenger RH, Stiehl DP, Camenisch G (2005). Integration of oxygen signaling at the consensus HRE. Sci. STKE.

[12] Patel SA, Simon MC (2008). Biology of hypoxia-inducible factor-2α in development and disease. Cell Death Differ..

[13] Dunwoodie SL (2009). The role of hypoxia in development of the mammalian embryo. Dev. Cell.

[14] Beall CM (2010). Natural selection on EPAS1 (HIF2α) associated with low hemoglobin concentration in Tibetan highlanders. Proc. Natl. Acad. Sci. USA.

[15] Pamenter ME, Hall JE, Tanabe Y, Simonson TS (2020). Cross-species insights into genomic adaptations to hypoxia. Front. Genet..

[16] Rytkönen KT, Williams TA, Renshaw GM, Primmer CR, Nikinmaa M (2011). Molecular evolution of the metazoan PHD-HIF oxygen-sensing system. Mol. Biol. Evol..

[17] Keith B, Johnson RS, Simon MC (2011). HIF1α and HIF2α: Sibling rivalry in hypoxic tumour growth and progression. Nat. Rev. Cancer.

[18] Storz JF (2021). High-altitude adaptation: Mechanistic insights from integrated genomics and physiology. Mol. Biol. Evol..

[19] Makino Y, Kanopka A, Wilson WJ, Tanaka H, Poellinger L (2002). Inhibitory PAS domain protein (IPAS) is a hypoxia-inducible splicing variant of the hypoxia-inducible factor-3α locus. J. Biol. Chem..

[20] Yang S-L, Wu C, Xiong Z-F, Fang X (2015). Progress on hypoxia-inducible factor-3: Its structure, gene regulation and biological function (Review). Mol. Med. Rep..

[21] Duan C (2016). Hypoxia-inducible factor 3 biology: Complexities and emerging themes. Am. J. Physiol. Cell Physiol..

[22] Nelson JS, Grande TC, Wilson MVH (2016). Fishes of the World.

[23] Mandic M, Joyce W, Perry SF (2021). The evolutionary and physiological significance of the Hif pathway in teleost fishes. J. Exp. Biol..

[24] Nikinmaa M, Rees BB (2005). Oxygen-dependent gene expression in fishes. Am. J. Physiol. Regul. Integr. Comp. Physiol..

[25] Pelster B, Egg M (2018). Hypoxia-inducible transcription factors in fish: Expression, function and interconnection with the circadian clock. J. Exp. Biol..

[26] Rytkönen KT (2013). Subfunctionalization of cyprinid hypoxia-inducible factors for roles in development and oxygen sensing. Evolution.

[27] Postlethwait JH (2000). Zebrafish comparative genomics and the origins of vertebrate chromosomes. Genome Res..

[28] Volff J-N (2005). Genome evolution and biodiversity in teleost fish. Heredity.

[29] Ohno S (1970). Evolution by Gene Duplication.

[30] Lynch M, Conery JS (2000). The evolutionary fate and consequences of duplicate genes. Science.

[31] Postlethwait JH (2007). The zebrafish genome in context: Ohnologs gone missing. J. Exp. Zool. B Mol. Dev. Evol..

[32] Graham AM, Presnell JS (2017). Hypoxia inducible factor (HIF) transcription factor family expansion, diversification, divergence and selection in eukaryotes. PLoS ONE.

[33] Berthelot C (2014). The rainbow trout genome provides novel insights into evolution after whole-genome duplication in vertebrates. Nat. Commun..

[34] Macqueen DJ, Johnston IA (2014). A well-constrained estimate for the timing of the salmonid whole genome duplication reveals major decoupling from species diversification. Proc. R. Soc. B Biol. Sci..

[35] Force A (1999). Preservation of duplicate genes by complementary, degenerative mutations. Genetics.

[36] Braasch I (2016). The spotted gar genome illuminates vertebrate evolution and facilitates human-teleost comparisons. Nat. Genet..

[37] Hughes LC (2018). Comprehensive phylogeny of ray-finned fishes (Actinopterygii) based on transcriptomic and genomic data. Proc. Natl. Acad. Sci. USA.

[38] Parey E (2020). Synteny-guided resolution of gene trees clarifies the functional impact of whole genome duplications. Mol. Biol. Evol..

[39] Townley IK (2017). Sequence and functional characterization of hypoxia-inducible factors, HIF1α, HIF2αa, and HIF3α, from the estuarine fish, *Fundulus heteroclitus*. Am. J. Physiol. Regul. Integr. Comp. Physiol..

[40] Gao F (2019). EasyCodeML: A visual tool for analysis of selection using CodeML. Ecol. Evol..

[41] Murrell B (2015). Gene-wide identification of episodic selection. Mol. Biol. Evol..

[42] Pond SLK (2011). A random effects branch-site model for detecting episodic diversifying selection. Mol. Biol. Evol..

[43] Smith MD (2015). Less is more: An adaptive branch-site random effects model for efficient detection of episodic diversifying selection. Mol. Biol. Evol..

[44] Murrell B (2012). Detecting individual sites subject to episodic diversifying selection. PLoS Genet..

[45] Pond SLK, Frost SDW (2005). Not so different after all: A comparison of methods for detecting amino acid sites under selection. Mol. Biol. Evol..

[46] Murrell B (2013). FUBAR: A fast, unconstrained bayesian approximation for inferring selection. Mol. Biol. Evol..

[47] Sandberg M, Eriksson L, Jonsson J, Sjostrom M, Wold S (1998). New chemical descriptors relevant for the design of biologically active peptides: A multivariate characterization of 87 amino acids. J. Med. Chem..

[48] Jombart T (2008). adegenet: A R package for the multivariate analysis of genetic markers. Bioinformatics.

[49] Jombart T, Ahmed I (2011). adegenet 1.3-1: New tools for the analysis of genome-wide SNP data. Bioinformatics.

[50] Wu D, Potluri N, Lu J, Kim Y, Rastinejad F (2015). Structural integration in hypoxia-inducible factors. Nature.

[51] Lando D (2002). FIH-1 is an asparaginyl hydroxylase enzyme that regulates the transcriptional activity of hypoxia-inducible factor. Genes Dev..

[52] Zhang P (2014). Hypoxia-inducible factor 3 is an oxygen-dependent transcription activator and regulates a distinct transcriptional response to hypoxia. Cell Rep..

[53] Gu Y-Z, Moran SM, Hogenesch JB, Wartman L, Bradfield CA (1998). Molecular characterization and chromosomal localization of a third α-class hypoxia inducible factor subunit, HIF3α. Gene Expr..

[54] Albanese A, Daly LA, Mennerich D, Kietzmann T, Sée V (2020). The role of hypoxia-inducible factor post-translational modifications in regulating its localisation, stability, and activity. Int. J. Mol. Sci..

[55] Daly LA (2021). Oxygen-dependent changes in binding partners and post-translational modifications regulate the abundance and activity of HIF-1α/2α. Sci. Signal..

[56] Geng H (2011). HDAC4 protein regulates HIF1α protein lysine acetylation and cancer cell response to hypoxia. J. Biol. Chem..

[57] Pasquier J (2016). Gene evolution and gene expression after whole genome duplication in fish: The PhyloFish database. BMC Genomics.

[58] Law SHW, Wu RSS, Ng PKS, Yu RMK, Kong RYC (2006). Cloning and expression analysis of two distinct HIF-alpha isoforms—gcHIF-1alpha and gcHIF-4alpha—from the hypoxia-tolerant grass carp, *Ctenopharyngodon idellus*. BMC Mol. Biol..

[59] Gasanov EV, Jędrychowska J, Kuźnicki K, Korzh V (2021). Evolutionary context can clarify gene names: Teleosts as a case study. BioEssays.

[60] Powell WH, Hahn ME (2002). Identification and functional characterization of hypoxia-inducible factor 2α from the estuarine teleost, *Fundulus heteroclitus*: Interaction of HIF-2α with two ARNT2 splice variants. J. Exp. Zool..

[61] Chi W, Gan X, Xiao W, Wang W, He S (2013). Different evolutionary patterns of hypoxia-inducible factor α (HIF-α) isoforms in the basal branches of Actinopterygii and Sarcopterygii. FEBS Open Bio.

[62] Guan L, Chi W, Xiao W, Chen L (2014). Analysis of hypoxia-inducible factor alpha polyploidization reveals adaptation to Tibetan plateau in the evolution of schizothoracine fish. BMC Evol. Biol..

[63] Chen J (2020). Analysis of multiplicity of hypoxia-inducible factors in the evolution of Triplophysa fish (Osteichthyes: Nemacheilinae) reveals hypoxic environments adaptation to Tibetan Plateau. Front. Genet..

[64] Wang Y (2015). Evidence for adaptation to the Tibetan Plateau inferred from Tibetan loach transcriptomes. Genome Biol. Evol..

[65] Rinaldi L (2005). Oxygen availability causes morphological changes and a different VEGF/FlK-1/HIF-2 expression pattern in sea bass gills. Ital. J. Zool..

[66] Rytkönen KT, Prokkola JM, Salonen V, Nikinmaa M (2014). Transcriptional divergence of the duplicated hypoxia-inducible factor alpha genes in zebrafish. Gene.

[67] Pan W (2022). Single-cell transcriptomic analysis of neuroepithelial cells and other cell types of the gills of zebrafish (*Danio rerio*) exposed to hypoxia. Sci. Rep..

[68] Macias D (2018). HIF-2α is essential for carotid body development and function. eLife.

[69] Kopp R, Köblitz L, Egg M, Pelster B (2011). HIF signaling and overall gene expression changes during hypoxia and prolonged exercise differ considerably. Physiol. Genomics.

[70] Reid NM (2016). The genomic landscape of rapid repeated evolutionary adaptation to toxic pollution in wild fish. Science.

[71] Cameron JN (1973). Oxygen dissociation and content of blood from Alaskan burbot (*Lota lota*), pike (*Esox lucius*) and grayline (*Thymallus arcticus*). Comp. Biochem. Physiol. Part A Physiol..

[72] Verberk WCEP (2022). Body mass and cell size shape the tolerance of fishes to low oxygen in a temperature-dependent manner. Glob. Change Biol..

[73] Katoh K, Standley DM (2013). MAFFT multiple sequence alignment software version 7: Improvements in performance and usability. Mol. Biol. Evol..

[74] Katoh K, Standley DM (2016). A simple method to control over-alignment in the MAFFT multiple sequence alignment program. Bioinformatics.

[75] Penn O (2010). GUIDANCE: A web server for assessing alignment confidence scores. Nucleic Acids Res..

[76] Sela I, Ashkenazy H, Katoh K, Pupko T (2015). GUIDANCE2: Accurate detection of unreliable alignment regions accounting for the uncertainty of multiple parameters. Nucleic Acids Res..

[77] Drummond AJ, Ho SYW, Phillips MJ, Rambaut A (2006). Relaxed phylogenetics and dating with confidence. PLoS Biol..

[78] Drummond AJ, Suchard MA, Xie D, Rambaut A (2012). Bayesian phylogenetics with BEAUti and the BEAST 1.7. Mol. Biol. Evol..

[79] Bouckaert R (2014). BEAST 2: A software platform for bayesian evolutionary analysis. PLoS Comput. Biol..

[80] Darriba D, Taboada GL, Doallo R, Posada D (2012). jModelTest 2: More models, new heuristics and parallel computing. Nat. Methods.

[81] Guindon S, Gascuel O (2003). A simple, fast, and accurate algorithm to estimate large phylogenies by maximum likelihood. Syst. Biol..

[82] Darriba D (2020). ModelTest-NG: A new and scalable tool for the selection of DNA and protein evolutionary models. Mol. Biol. Evol..

[83] Flouri T (2015). The phylogenetic likelihood library. Syst. Biol..

[84] Jones DT, Taylor WR, Thornton JM (1992). The rapid generation of mutation data matrices from protein sequences. Bioinformatics.

[85] Drummond AJ, Rambaut A (2007). BEAST: Bayesian evolutionary analysis by sampling trees. BMC Evol. Biol..

[86] Kumar S, Stecher G, Li M, Knyaz C, Tamura K (2018). MEGA X: Molecular evolutionary genetics analysis across computing platforms. Mol. Biol. Evol..

[87] Felsenstein J (1985). Confidence limits on phylogenies: An approach using the bootstrap. Evolution.

[88] Thompson JD, Higgins DG, Gibson TJ (1994). CLUSTAL W: Improving the sensitivity of progressive multiple sequence alignment through sequence weighting, position-specific gap penalties and weight matrix choice. Nucleic Acids Res..

[89] Henikoff S, Henikoff JG (1992). Amino acid substitution matrices from protein blocks. Proc. Natl. Acad. Sci. USA.

[90] Stamatakis A (2014). RAxML version 8: A tool for phylogenetic analysis and post-analysis of large phylogenies. Bioinformatics.

[91] Miller, M. A., Pfeiffer, W. & Schwartz, T. Creating the CIPRES Science Gateway for inference of large phylogenetic trees. In *Proceedings of the Gateway Computing Environments Workshop (GCE)* 1–8 (Institute of Electrical and Electronics Engineers, 2010). 10.1109/GCE.2010.5676129.

[92] Ranwez V, Harispe S, Delsuc F, Douzery EJP (2011). MACSE: Multiple Alignment of Coding SEquences accounting for frameshifts and stop codons. PLoS ONE.

[93] Anisimova M, Bielawski JP, Yang Z (2001). Accuracy and power of the likelihood ratio test in detecting adaptive molecular evolution. Mol. Biol. Evol..

[94] Yang Z (1998). Likelihood ratio tests for detecting positive selection and application to primate lysozyme evolution. Mol. Biol. Evol..

[95] Pond SLK, Frost SDW, Muse SV (2005). HyPhy: Hypothesis testing using phylogenies. Bioinformatics.

[96] Pond SL (2020). HyPhy 2.5—A customizable platform for evolutionary hypothesis testing using phylogenies. Mol. Biol. Evol..

[97] Pond SLK, Frost SDW (2005). Datamonkey: Rapid detection of selective pressure on individual sites of codon alignments. Bioinformatics.

[98] Delport W, Poon AFY, Frost SDW, Pond SLK (2010). Datamonkey 2010: A suite of phylogenetic analysis tools for evolutionary biology. Bioinformatics.

[99] Weaver S (2018). Datamonkey 2.0: A modern web application for characterizing selective and other evolutionary processes. Mol. Biol. Evol..

[100] RStudio Team. *RStudio: Integrated Development for R* (2022).

[101] Webb B, Sali A (2016). Comparative protein structure modeling using MODELLER. Curr. Protoc. Bioinform..

[102] Waterhouse AM, Procter JB, Martin DMA, Clamp M, Barton GJ (2009). Jalview Version 2—A multiple sequence alignment editor and analysis workbench. Bioinformatics.

[103] Tarade D, Lee JE, Ohh M (2019). Evolution of metazoan oxygen-sensing involved a conserved divergence of VHL affinity for HIF1α and HIF2α. Nat. Commun..

[104] Zhao S, Ye Z, Stanton R (2020). Misuse of RPKM or TPM normalization when comparing across samples and sequencing protocols. RNA.

[105] Townley, I. K., Babin, C., Murphy, T. E., Summa, C. M. & Rees, B. Evolution of HIFA Actinopterygii Datasets. 10.6084/m9.figshare.21713759.v1 (2022).

