# Supplementary material for: Genomic analysis of hypoxia inducible factor alpha in ray-finned fishes reveals missing Ohnologs and evidence of widespread positive selection
Source: Sci Rep. 2022 Dec 24;12:22312. doi: 10.1038/s41598-022-26876-7 (PMC9789988; doi:10.1038/s41598-022-26876-7)

Fig. S1. Phylogeny of Actinopterygian *HIFA* reconstructed by Bayesian inference using full-length CDS. Evolutionary analyses were conducted in BEAST 2 (v2.6.1) using the general time reversible model (GTR) with six gamma categories (+G) and allowing for invariants (+I). The tree with maximum clade credibility and mean heights is shown with posterior probability values next to the branches. The highest tree likelihood was -41437.47 with an ESS of 587. The tree was re-rooted on the outgroup for visualization. Four *HIFA* homology groups are indicated to the right and the following taxa are color coded within each group: Otocephala (green); Salmoniformes (orange); Neoteleostei (blue). The outgroup, *Ciona intestinalis*, basal Actinopterygian (spotted gar, *Lepisosteus oculatus*), basal teleost (Asian arowana, *Scleropages formosus*), and sister taxa to Salmoniformes (Northern pike, *Esox lucius*), are not color coded. Sequences are identified by the first letter of the genus and species followed by the last four digits of the NCBI or Ensemble reference gene accession number (see supplementary table S1 for a full list of genes). **NOTE:** this is a rectangular representation of the tree as shown in text Fig. 1 in circular format.

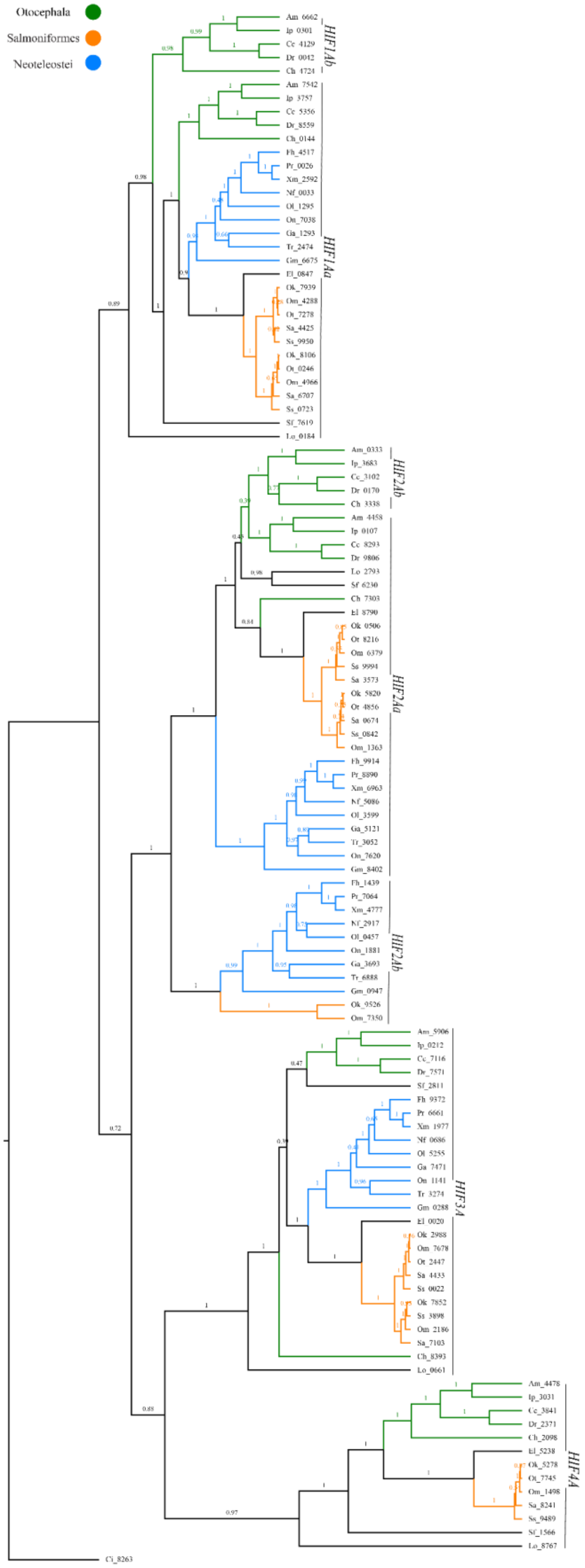

Fig. S1. Phylogeny of Actinopterygian *HIFA* reconstructed by Bayesian inference using full-length CDS. Evolutionary analyses were conducted in BEAST 2 (v2.6.1) using the general time reversible model (GTR) with six gamma categories (+G) and allowing for invariants (+I). The tree with maximum clade credibility and mean heights is shown with posterior probability values next to the branches. The highest tree likelihood was -41437.47 with an ESS of 587. The tree was re-rooted on the outgroup for visualization. Four *HIFA* homology groups are indicated to the right and the following taxa are color coded within each group: Otocephala (green); Salmoniformes (orange); Neoteleostei (blue). The outgroup, *Ciona intestinalis*, basal Actinopterygian (spotted gar, *Lepisosteus oculatus*), basal teleost (Asian arowana, *Scleropages formosus*), and sister taxa to Salmoniformes (Northern pike, *Esox lucius*), are not color coded. Sequences are identified by the first letter of the genus and species followed by the last four digits of the NCBI or Ensemble reference gene accession number (see supplementary table S1 for a full list of genes). **NOTE:** this is a rectangular representation of the tree as shown in text Fig. 1 in circular format.

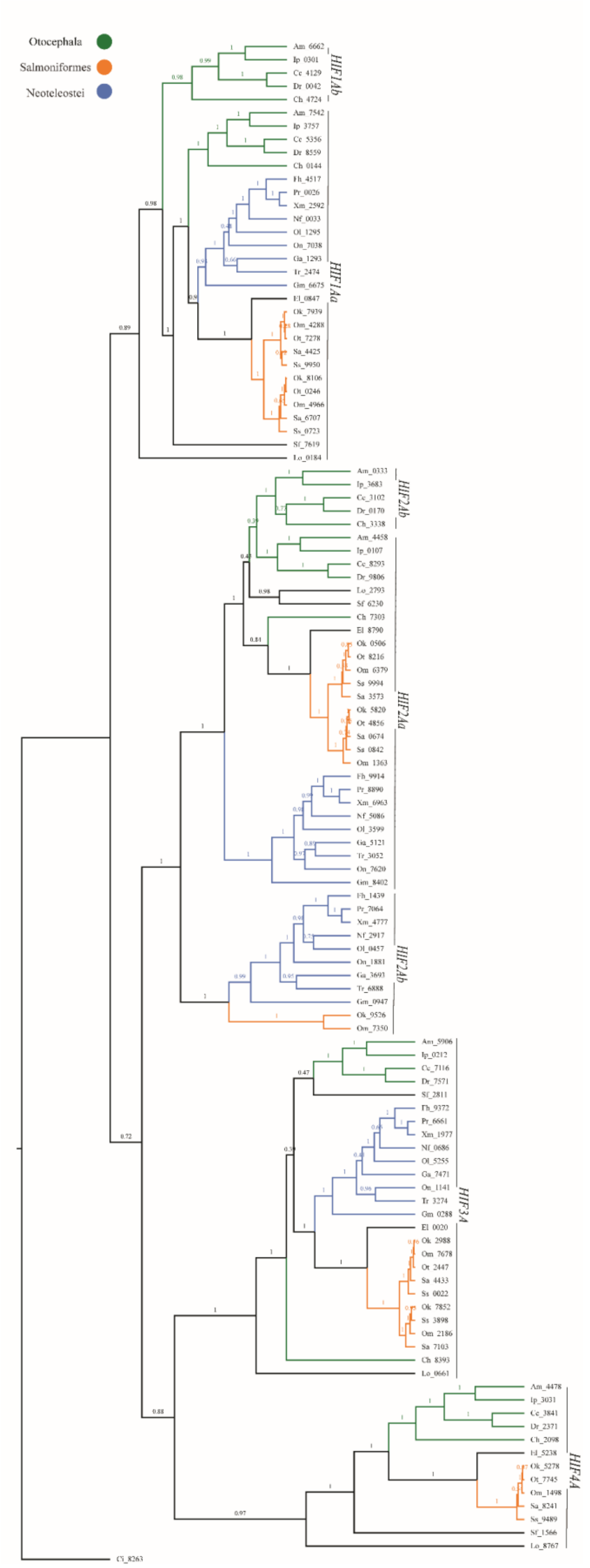

Fig. S3. Phylogeny of Actinopterygian *HIFA* reconstructed by Bayesian inference using amino acid sequences deduced for full length proteins. Evolutionary analyses were conducted in BEAST 2 (v2.6.1) using a JTT matrix-based model with six gamma categories (+G) and allowing for invariants (+I). The tree with maximum clade credibility and mean heights is shown with posterior probability values next to the branches. The highest tree likelihood was -20378.55 with an ESS of 964.The tree was re-rooted on the outgroup for visualization. Four *HIFA* homology groups are indicated to the right and the following taxa are color coded within each group: Otocephala (green); Salmoniformes (orange); Neoteleostei (blue). The outgroup, *Ciona intestinalis*, basal Actinopterygian (spotted gar, *Lepisosteus oculatus*), basal teleost (Asian arowana, *Scleropages formosus*), and sister taxa to Salmoniformes (Northern pike, *Esox lucius*), are not color coded. Sequences are identified by the first letter of the genus and species followed by the last four digits of the NCBI or Ensemble reference gene accession number (see supplementary table S1 for a full list of genes).

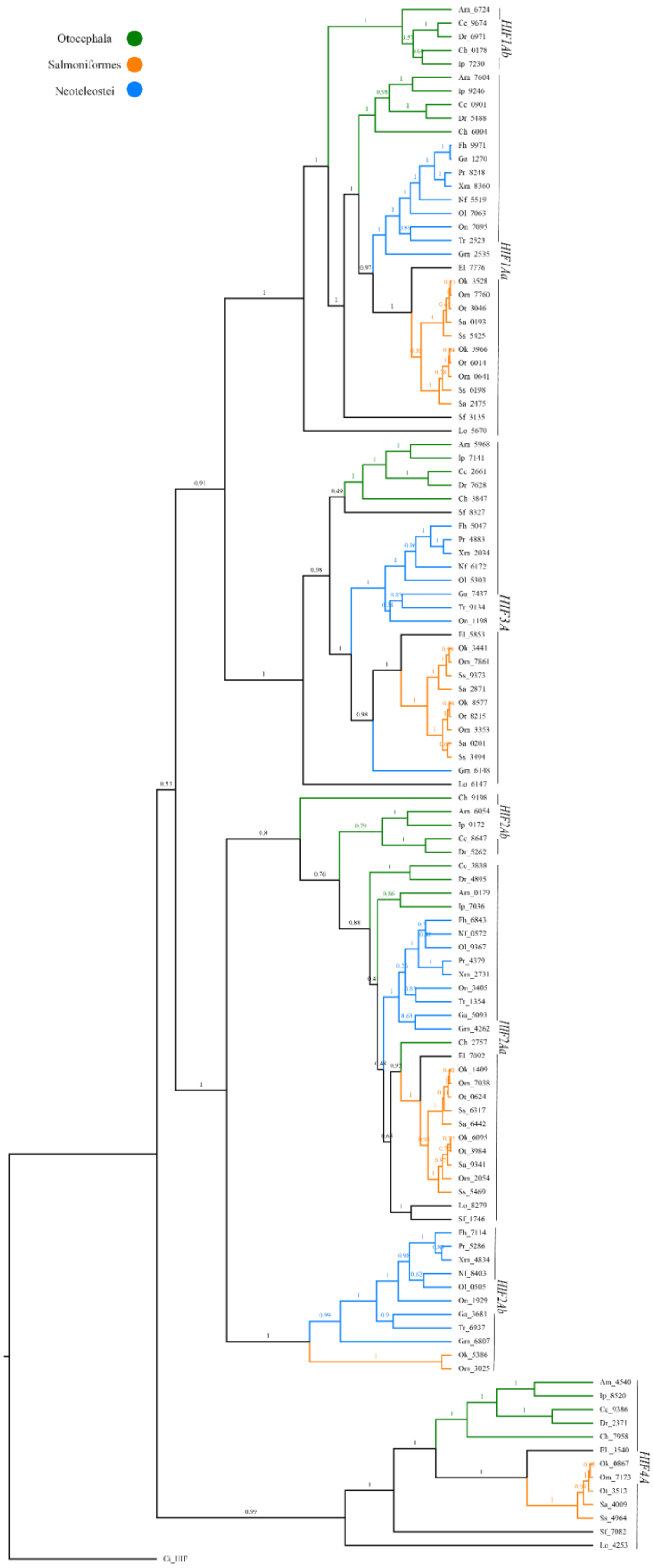

Fig. S4. Phylogeny of Actinopterygian *HIFA* reconstructed by maximum likelihood using amino acid sequences deduced for full length proteins. Evolutionary analyses were conducted in MEGAX (v10.1.8) using a JTT matrix-based model with six gamma categories (+G) and allowing for invariants (+I). The tree with the highest log likelihood (-20259.51) is shown with bootstrap values next to the branches. The tree was re-rooted on the outgroup after analysis. Four *HIFA* homology groups are indicated to the right and the following taxa are color coded within each group: Otocephala (green); Salmoniformes (orange); Neoteleostei (blue). The outgroup, *Ciona intestinalis*, basal Actinopterygian (spotted gar, *Lepisosteus oculatus*), basal teleost (Asian arowana, *Scleropages formosus*), and sister taxa to Salmoniformes (Northern pike, *Esox lucius*), are not color coded. Sequences are identified by the first letter of the genus and species followed by the last four digits of the NCBI or Ensemble reference gene accession number (see supplementary table S1 for a full list of genes).

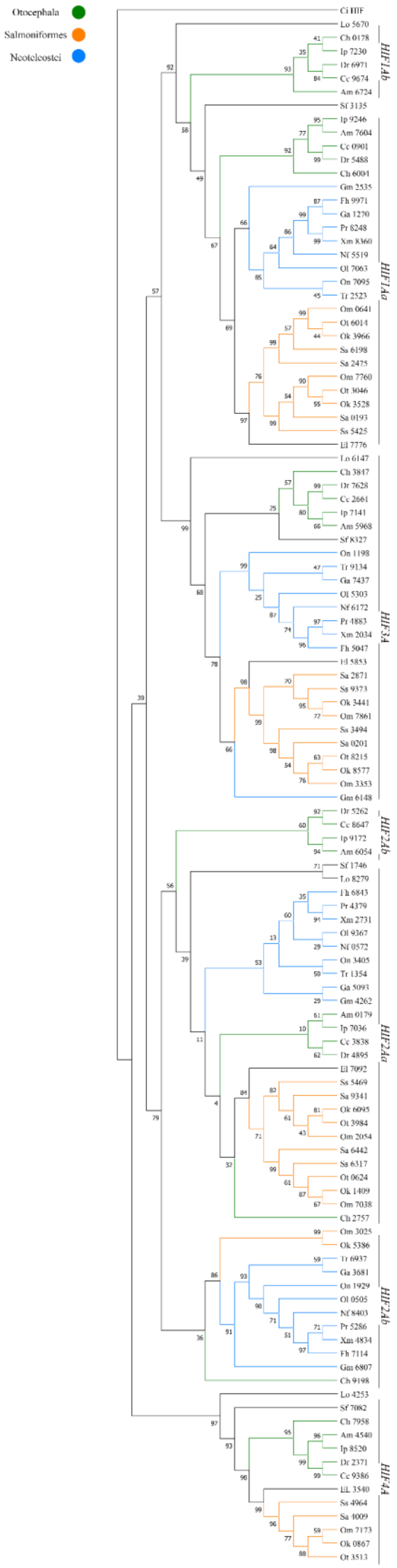

Fig. S5. *HIFA* paralogs grouped according to the physiochemical properties of amino acids potentially under positive selection. a. *HIF1A* paralogs were placed in three groups: (1) *HIF1A* from spotted gar (*Lepisosteus oculatus*) and *HIF1Aa* from Otocephala (2) *HIF1Aa* from Asian arowana (*Scleropages formosus*) and Otocephala *HIF1Ab* except Cc_4129, and (3) *HIF1Aa* from Salmoniformes and sister taxa Northern pike (*Esox lucius*), all Neoteleostei, and Cc_4129. **b.** Analysis of *HIF2A* paralogs grouped the truncated forms of *HIF2Ab* together. The remaining paralogs were placed in three groups: (1) *HIF2Aa* from all Actinopterygii except Cc_8293, Om_6379, and Pr_8890, (2) Otocephala *HIF2Ab*, and (3) *HIF2Aa* Cc_8293, Om_6379, and Pr_8890. **c.** *HIF3A* paralogs were placed in two groups: (1) *HIF3A* from all Actinopterygii except Salmoniformes and (2) *HIF3A* from Salmoniformes. **d.** *HIF4A* paralogs were placed in two groups: (1) *HIF4A* from Asian arowana, Otocephala and Northern pike and (2) *HIF4A* from Salmoniformes (see supplementary table S3 for individual amino acids at each site for each paralog).

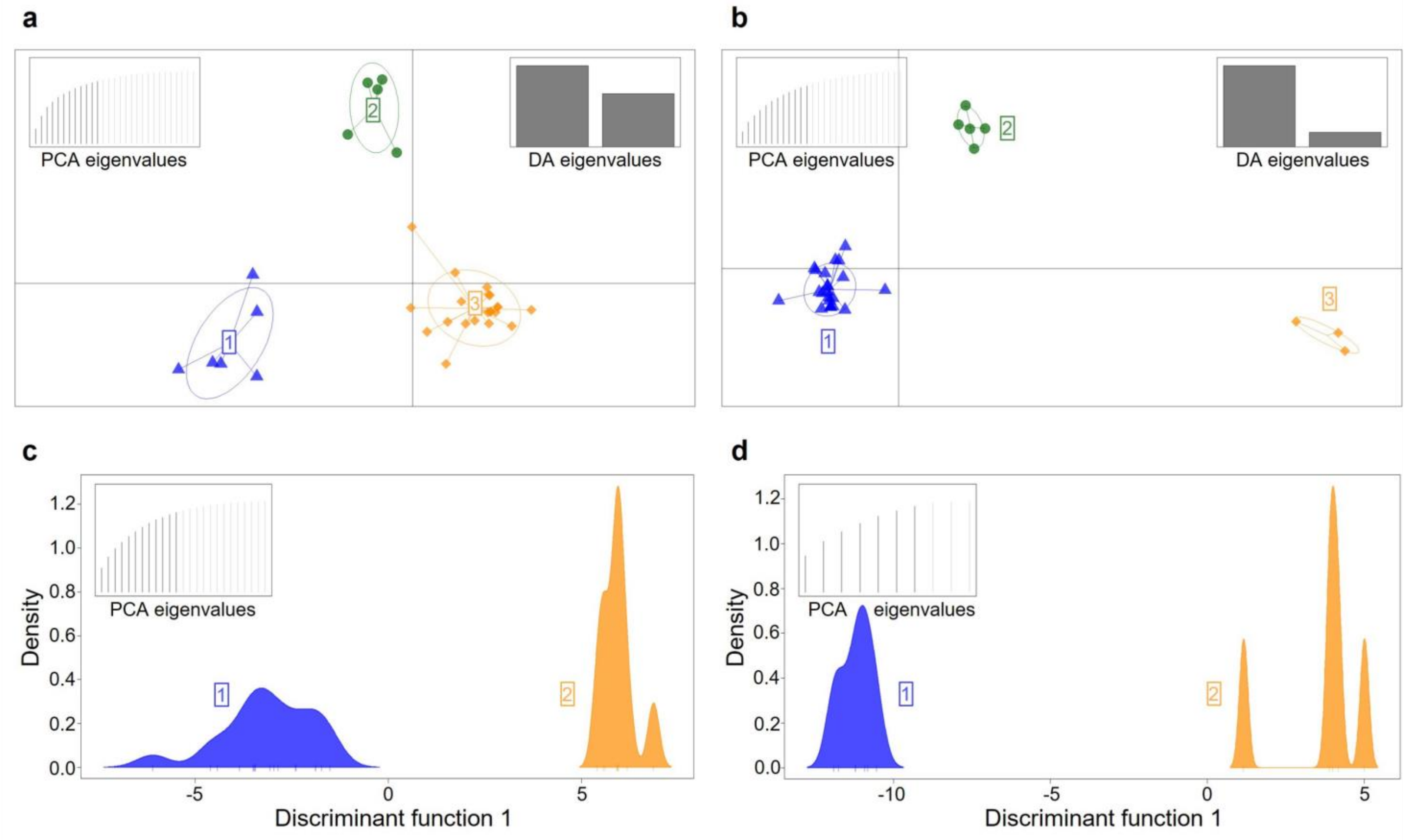

Fig. S6. Structural model of the amino-terminal half of actinopterygian HIF1α showing sites putatively under positive selection. Five residues potentially under positive selection (MSA Codons 65-484, Table 3) mapped to a structural model of the N-terminal half of HIF1α (orange spheres). The upper and lower images are rotated by 180° around the indicated plane. Conserved DNA-binding (bHLH) and protein dimerization (PAS-A, PAS-B) domains are indicated at the top.

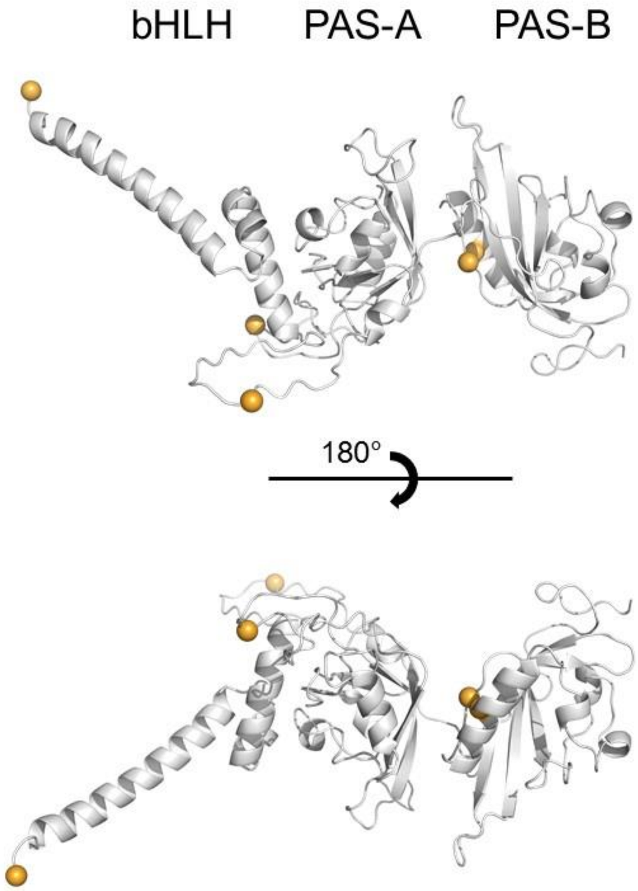

Supplement: Supplementary file 5 — Supplementary Figures. [file 41598_2022_26876_MOESM5_ESM.pdf]
